# Aging Disrupts L-type Ca^2+^ Channel Organization and Function in Pacemaker Cells

**DOI:** 10.1161/CIRCRESAHA.125.327894

**Published:** 2026-06-19

**Authors:** Oscar Vivas, Matthias Baudot, Roxanne Madden, Wendy L. Piñon-Teal, Martina S. Hunt, Sabrina Choi, Roya Pournejati, Victor A. Flores-Tamez, L. Fernando Santana, Claudia M. Moreno

**Affiliations:** 1 Department of Pharmacology (O.V., R.P.), University of Washington, Seattle.; 2 Department of Neurobiology and Biophysics (O.V., M.B., R.M., W.L.P.-T., M.S.H., S.C., C.M.M.), University of Washington, Seattle.; 3 Department of Pharmacology (V.A.F.-T), University of California, Davis.; 4 Department of Physiology and Membrane Biology (L.F.S.), University of California, Davis.; 5 Howard Hughes Medical Institute, Chevy Chase, MD (C.M.M.).

**Keywords:** aging, calcium channels, caveolae, cell membrane, heart rate, sinoatrial node

## Abstract

**BACKGROUND:**

Every heartbeat is initiated by a spontaneous electrical signal generated inside the cardiac pacemaker. This signal depends on the coordinated activity of ion channels, where voltage-gated L-type calcium channels play a central role. All mammals experience a progressive decline in pacemaker rate with age, which in humans can become pathological and drive the need for artificial pacemaker implantation. Yet the mechanisms underlying this age-associated slowdown remain incompletely understood.

**METHODS:**

We combined patch-clamp electrophysiology, single-channel recording, calcium imaging, immunocytochemistry, and super-resolution microscopy to investigate L-type calcium channel density, organization, and function in sinoatrial node pacemaker cells from young and old mice.

**RESULTS:**

Aging reduced L-type calcium channel density at the plasma membrane, disrupted channel clustering, and decreased open probability, collectively diminishing calcium current density by 50%. Pharmacological enhancement of channel open probability with Bay K 8644 was sufficient to accelerate diastolic depolarization and restore pacemaker rate in old cells to young levels. These channel alterations were paralleled by a decline in caveolin-3 expression. Disrupting caveolae in young cells recapitulated the loss of channel clustering, pointing to caveolae as a structural basis for the age-associated channel alterations.

**CONCLUSIONS:**

Aging impairs L-type calcium channel organization and function in cardiac pacemaker cells. This impairment is mediated by an age-associated reduction of caveolae, key membrane microdomains for channel organization. The resulting decline in L-type calcium channel density and activity is an important driver of the age-associated slowdown of the cardiac pacemaker.

**Novelty and Significance**

**What Is Known?**

- Every heartbeat is triggered by a spontaneous and rhythmic electrical signal fired by the sinoatrial node pacemaker.
- L-type calcium channels are fundamental for regenerating the depolarization that triggers each new beat and for sustaining the pacemaker action potential.
- With age, mammals experience a progressive decline in the pacemaker rate, but the mechanisms remain incompletely understood.

**What New Information Does This Article Contribute?**

- Aging reduces L-type calcium channel surface density, disrupts channel clustering, and decreases open probability, collectively diminishing calcium current density by 50% in pacemaker cells.
- These changes parallel a decline in caveolin-3; moreover, disrupting caveolae in young cells recapitulates the loss of channel clustering, linking channel disorganization to the loss of caveolae, the microdomains that spatially organize these channels.
- Enhancing L-type channel open probability with Bay K 8644 is sufficient to accelerate diastolic depolarization and restore the firing rate of old pacemaker cells to young levels.

The cardiac pacemaker dictates how fast the heart beats, and this pace slows with age. The slowing can become pathological, making it a leading reason for pacemaker implantation in older adults. Why pacemaker cells slow with age has remained unclear. We show that pacemaker cells from old mice exhibit a disruption in the organization and function of L-type calcium channels. Aging reduces L-type calcium channel density at the plasma membrane, clustering, and activity, collectively diminishing calcium current density by 50% and contributing to the slowdown of firing rate. These changes are paralleled by a decline in caveolin-3, and disrupting caveolae in young cells recapitulates the loss of clustering, pointing to caveolae as the structural basis for the channel alterations. Enhancing channel open probability with Bay K 8644 accelerates diastolic depolarization and restores the firing rate of old cells to young levels, showing that the slowing is reversible at the channel level. Together, these findings link the slowing pacemaker to the loss of caveolae and the resulting reduction and disorganization of L-type calcium channels, and point more broadly to how age-related changes in plasma-membrane organization can alter cellular electrical activity.

Mammals, including humans and mice, experience a linear decline in heart rate with age.^1–5^ How fast the heart beats depends on the activity of the cardiac pacemaker, a specialized region of the heart that fires spontaneous and rhythmic action potentials. The electrical signal originating inside the pacemaker propagates through the electrical conduction system of the heart, triggering the depolarization and contraction of the atrial and ventricular chambers. This cycle is repeated without interruption from embryonic development until death. The intrinsic pacemaker rate declines linearly from birth at a rate of ≈0.8 bpm/y in humans and ≈4 bpm/mo in mice.^1^ This natural slowdown of the pacemaker can become pathological. In fact, the age-associated slowdown of the pacemaker is the main cause of the more than half a million artificial pacemaker devices implanted annually worldwide.^6,7^ However, the molecular mechanisms behind the slowdown of the pacemaker are not completely understood.

The automaticity of the pacemaker relies on a fine balance of ionic currents that drive and allow the regeneration of the pacemaker action potential, where voltage-gated L-type calcium channels play a key role. The pacemaking mechanism can be seen as an oscillatory cycle of the membrane potential. Although oscillatory in nature, the cycle could be described starting in the diastolic depolarization (DD) phase, which can be divided in Early and Late DD. The Early DD involves the activation of hyperpolarization-activated cyclic nucleotide-gated channel isoform 4 (HCN4) channels^8,9^ and T-type voltage-gated calcium channel α1G subunit^10^ at voltages below −60 mV. The subsequent Late DD starts at around −55 mV with the opening of the L-type voltage-gated calcium channel α1D subunit (Ca_V_1.3) channels.^10^ This inward current depolarizes the membrane further enough to reach the action potential threshold, around −40 mV, where L-type α1C subunit (Ca_V_1.2) voltage-gated calcium channels activate. The pacemaker action potential is a calcium action potential mainly carried by Ca_V_1.2 and Ca_V_1.3 L-type calcium channels. The cycle is completed by the inactivation of calcium channels and the activation of several potassium channels, including K_V_4.2/4.3 (I_to_), K_V_1.5 (I_Kur_), hERG (I_Kr_), and K_V_7.1/KCNE (I_Ks_), resulting in the repolarization phase of the action potential.^11^ Repolarization leads to the activation of the DD, which starts the cycle again.

L-type calcium channels play an essential role not only in the generation of each action potential but also in the control of firing rate. Knockout mice lacking the Ca_V_1.3 channel exhibit bradycardia and sinus pauses.^12,13^ In addition, humans carrying point mutations that reduce the function of Ca_V_1.3 channels also exhibit bradycardia,^14,15^ resembling the pacemaker dysfunction observed in the elderly. The remodeling of ionic currents, including the downregulation or upregulation of the expression of specific ion channels, has been proposed to be one of the primary mechanisms underlying the age-associated dysfunction of the cardiac pacemaker.^16^ The best-understood change is the alteration of HCN4 channels. HCN4 current density is reduced, and the voltage-dependent activation is shifted in aged animals.^1,17,18^ The age-associated alteration of HCN4 is not controversial. However, how aging alters L-type calcium channels is still under debate.

Support and opposition for age-associated changes in L-type calcium channels have been documented. Ca_V_1.2 mRNA increases slightly, whereas Ca_V_1.3 mRNA does not change in aged rats.^16^ At the protein level, the expression of Ca_V_1.2 channels in the pacemaker has been reported to be reduced by about 40% in aged rats,^19^ and almost 80% in aged guinea pigs.^20^ In aged mouse pacemaker cells, L-type calcium current density is reduced; however, this reduction was attributed to cell hypertrophy rather than to a change in channel number or function.^1^ An in silico model concluded that the remodeling of L-type calcium current (I_CaL_), either via a gain or loss of function, is predicted to be the most significant factor underlying the slowing down of cardiac pacemaking rates in old animals.^21^ Together, these studies leave several questions open: is the reduction in L-type calcium current a consequence of cell hypertrophy, or does it reflect molecular changes to the channels themselves? Do channels at the plasma membrane change in density, organization, or gating? And what is the molecular mechanism connecting aging to such changes?

Here, we combined electrophysiology, super-resolution microscopy, and pharmacology to characterize L-type calcium channel expression, density, organization, and function in sinoatrial node pacemaker cells isolated from young and old mice. Our results provide evidence that aging leads to a reduction in the density, clustering, and activity of L-type calcium channels, contributing to the pacemaker’s age-associated slowdown.

## Methods

### Data Availability

A detailed description of all experimental procedures, including pacemaker cell isolation, electrophysiology, immunohistochemistry, super-resolution imaging, calcium photometry, and biochemical assays, is provided in the Expanded Materials and Methods section of the Supplemental Material. That section also includes a description of the study design and statistical analyses used. Specific statistical tests and sample sizes are indicated in the figure legends. A Major Resources Table listing all antibodies, chemicals, software, and animal models used in this study is also included in the Supplemental Material. All data have been deposited and are accessible at Dryad (https://doi.org/10.5061/dryad.sn02v6xmb).

## Results

### Sinoatrial Node and Pacemaker Cell Identification

**Figure 1.**
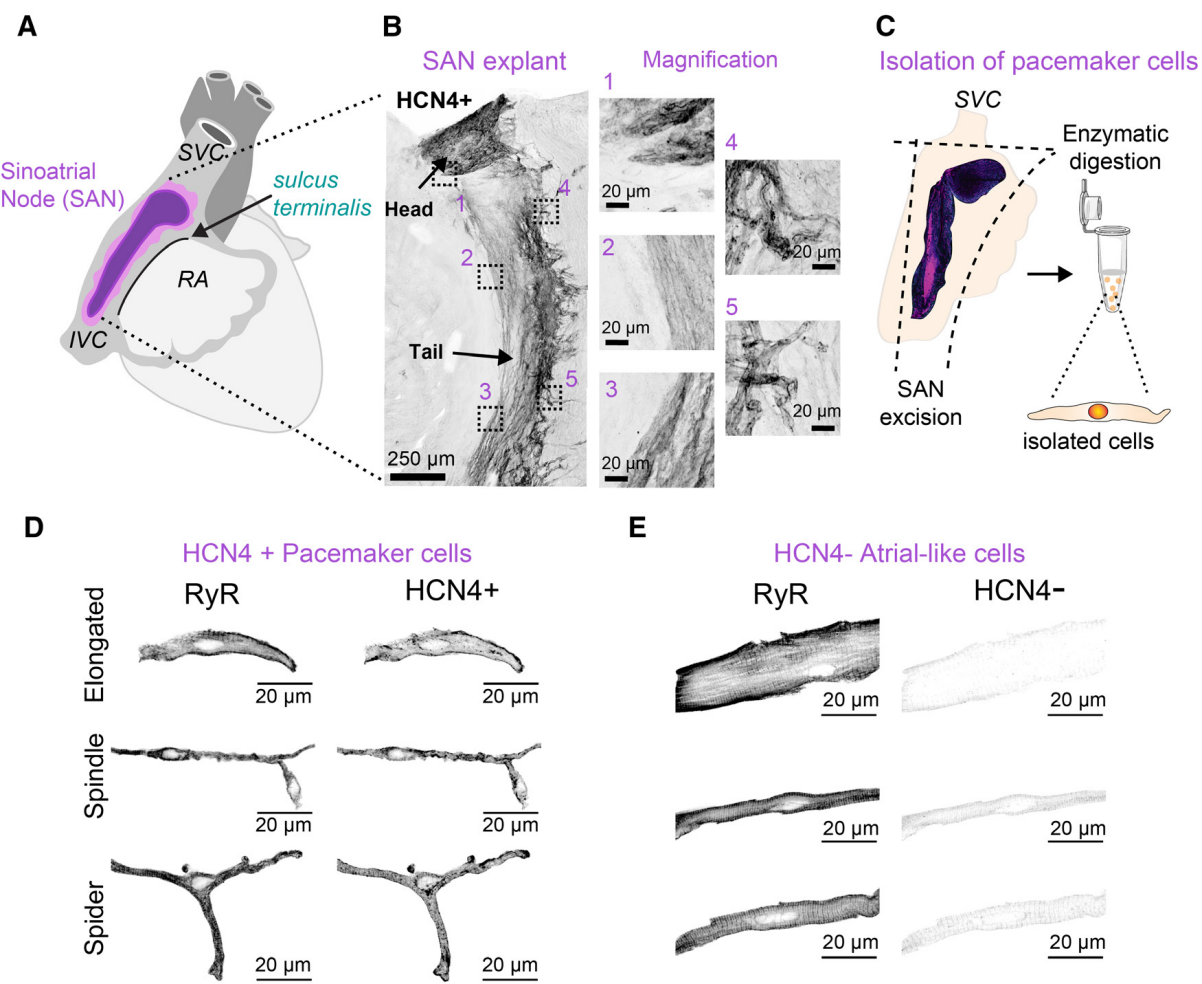
**Identification of the sinoatrial node and pacemaker cells. A**, Schematic of the sinoatrial node (SAN) location, between the right atrium (RA) and the superior vena cava (SVC) and inferior vena cava (IVC). **B**, Volumetric reconstruction of a cleared SAN explant immunostained against hyperpolarization-activated cyclic nucleotide-gated channel isoform 4 (HCN4), with head and tail regions identified. **Right**: magnification of 5 regions showing cellular organization. **C**, Schematic of the dissection and isolation of single pacemaker cells. **D** and **E**, Isolated cells stained against HCN4 and ryanodine receptor type 2 (RyR2). **D**: 3 representative HCN4-positive pacemaker cells. **E**: 3 representative HCN4-negative atrial-like cells.

We studied the sinoatrial node pacemaker, as illustrated in Figure 1A. The sinoatrial node is delimited at the top by the superior vena cava, at the right by the sulcus terminalis in the right atrium, and at the bottom by the coronary sulcus and the inferior vena cava. As shown in Figure 1B, the sinoatrial pacemaker is organized into defined head and tail structures. For live-cell isolation, this same region was excised and processed enzymatically (Figure 1C). Cell identity after isolation was also confirmed using immunostaining against HCN4. As shown in Figure 1D, besides HCN4-positive staining, pacemaker cells can be easily identified by their small and thin size compared with nonpacemaker cells. In addition, pacemaker cells exhibit 3 different morphologies: spindle, elongated, and spider, as other groups have reported,^22,23^ which are easily distinguishable from large, square HCN4-negative cells (Figure 1E). Another useful criterion for identifying pacemaker cells is that they exhibit less prominent striations, which can be observed with bright-field illumination and are more evident when cells are stained against ryanodine receptor type 2 (Figure 1D and 1E). For fixed-cell experiments, pacemaker identity was corroborated by HCN4 coimmunostaining; only HCN4-positive cells were included in our analyses. For live-cell experiments, cells were selected based on their morphology (including spider, spindle, and elongated cells) and striation pattern.

### Aging Slows Down the Firing Rate and the DD of Pacemaker Cells

**Figure 2.**
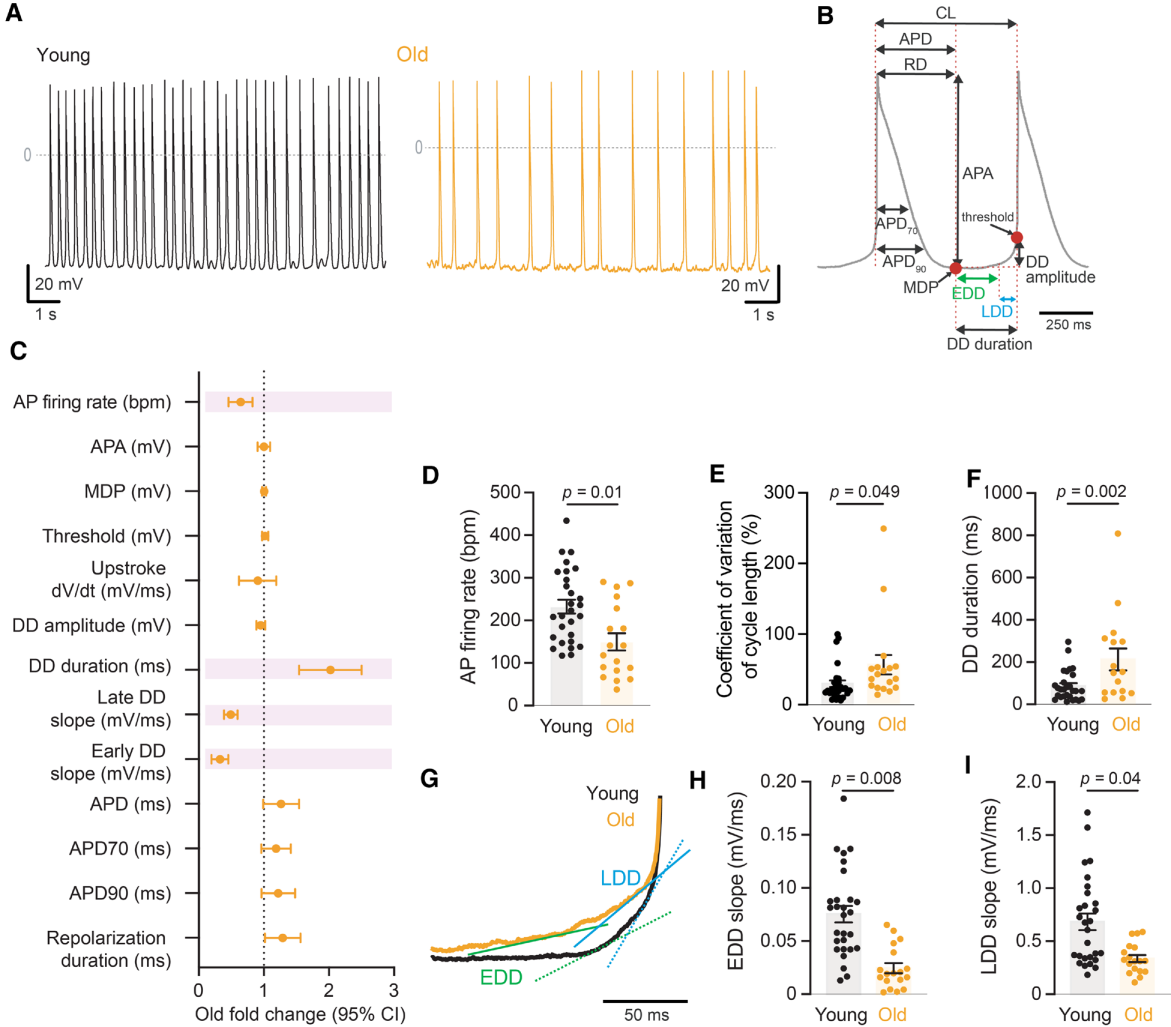
**Aging slows the spontaneous firing rate and the diastolic depolarization of pacemaker cells. A**, Representative spontaneous action potentials from young (black) and old (orange) pacemaker cells. **B**, Schematic of 2 pacemaker action potentials defining the analyzed parameters. **C**, Fold change of action potential parameters in old vs young cells; vertical dotted line represents the young population; purple bars indicate significantly different parameters. **D**, Firing rate, young vs old. **E**, Coefficient of variation (CV) of cycle length, young vs old. **F**, Diastolic depolarization (DD) duration, young vs old. **G**, Representative DD traces from a young and old cell, with fitted lines for early diastolic depolarization (EDD; green) and late diastolic depolarization (LDD; blue) slopes. **H** and **I**, EDD and LDD slopes, young vs old. Bars are mean±SEM. Statistical comparisons used a nested *t* test to account for cells nested within animals (young n=28 cells from 5 mice; old n=18 cells from 5 mice). APA indicates action potential amplitude; APD, action potential duration; CL, cycle length; MDP, maximum diastolic potential; and RD, repolarization duration.

We recorded the spontaneous electrical activity of isolated pacemaker cells from 4- to 6- and 24- to 30-month-old mice (Figure 2A). These ages are equivalent to 23 to 30 and 69 to 81 years in humans.^24^ Recordings were performed between 32 °C to 34 °C and only spontaneously firing cells with capacitance below 50 pF were analyzed. Figure 2B illustrates the parameters measured from the action potential waveforms to compare young and old cells. Of all the analyzed parameters, significant changes in the old group were found for the action potential firing rate, the DD duration, and the slopes of the early DD (EDD) and late DD (LDD; Figure 2C). At the population level, the action potential firing rate was significantly slower in old cells, with a mean±SEM value of 150±20 bpm compared with 232±16 bpm in young cells (Figure 2D). The firing rate from young animals was comparable to that reported by Marger et al^25^ (average of 260 bpm at 36 °C). The beat-to-beat mean cycle length was 295 ms (95% CI, 253–337) for young cells and 543 ms (95% CI, 376–711) for old cells. Old cells also showed greater rhythm irregularity, with a higher SD of cycle length (375±127 ms versus 87±14 ms in young cells) and a higher coefficient of variation (56.6% versus 29.6% in young cells; Figure 2E), indicating that the increased irregularity is not solely due to slower firing. Since the DD duration and rate of depolarization (ie, slope) are key determinants of how fast pacemaker cells can fire, we evaluated the differences between these parameters in young and old cells. Old cells showed a longer DD duration, with an average of 199±24 ms compared with 106±9 ms for the young cells (Figure 2F). Figure 2G exemplifies the flattening of the early DD and late DD slopes observed in old pacemaker cells. We found that old cells have a significant reduction in the slopes of both EDD (young, 0.075±0.007 mV/ms versus old, 0.024±0.004 mV/ms; Figure 2H) and LDD (young, 0.68±0.08 mV/ms versus old, 0.34±0.03 mV/ms; Figure 2I).

### L-Type Calcium Current Density Is Reduced in Old Pacemaker Cells

**Figure 3.**
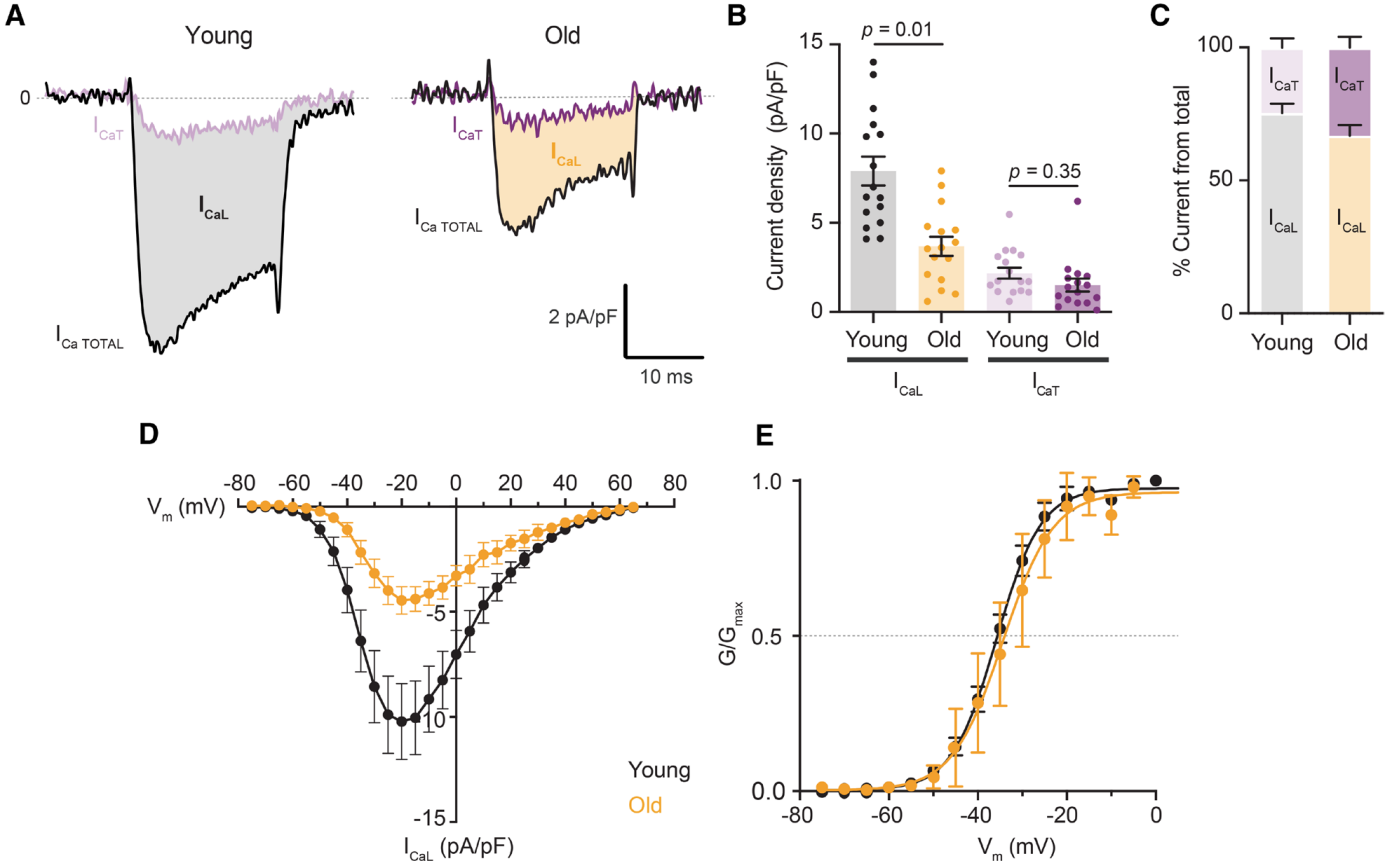
**L-type calcium current density is reduced in old pacemaker cells. A**, Representative recordings of total calcium current (I_Ca TOTAL_, black) and the nifedipine-resistant component (T-type calcium channel [I_CaT_], light/dark purple) from young and old cells. Shaded region: nifedipine-sensitive component (I_CaL_; gray, young; orange, old). **B**, L- and T-type current density, young vs old. **C**, Percentages of I_CaL_ and I_CaT_ relative to total calcium current in each age group. Total calcium current in old cells is also shown as 100% but is only 50% of the young value. **D**, I_CaL_ current-voltage relationship, young vs old. **E**, Normalized conductance (G)–voltage relationship for I_CaL_, young vs old. Bars are mean±SEM (young n=16 cells from 4 mice; old n=16 cells from 4 mice). Statistical comparisons for **B** used 2 independent nested *t* tests (young vs old for I_CaL_; young vs old for I_CaT_).

The flattening of the DD observed in old pacemaker cells suggests that aging affects the ion channels responsible for this spontaneous depolarization. We focused on L-type calcium channels, because mutations in Ca_V_1.3 cause bradycardia similar to that observed in the elderly.^14,15^ To determine whether isolated pacemaker cells from young and old animals differed in their calcium current density, we recorded whole-cell calcium currents from isolated pacemaker cells. Figure 3A shows representative traces of the total calcium current in young and old pacemaker cells. To isolate I_CaL,_ we used 10 μM nifedipine, a concentration that selectively blocks L-type channels without affecting the T-type voltage-gated calcium channel α1G subunit (IC_50_=109 μM). As shown in the light and dark purple traces in Figure 3A, only a small component of the total calcium current is resistant to nifedipine and, hence, carried by T-type calcium channels. The analysis of the I_CaL_ and T-type calcium channel current densities showed that aging reduced the I_CaL_ by 53%, going from 7.9±0.8 pA/pF in young cells to 3.7±0.5 pA/pF in the old cells (Figure 3B). In contrast, no statistically significant difference in T-type calcium channel was detected, with values of 2.2±0.3 pA/pF in young cells and 1.5±0.4 pA/pF in old cells. The percentage of total calcium current carried by each component is shifted with age, with a larger contribution of T-type in old cells (Figure 3C). We also evaluated the effect of aging on the voltage dependence of the L-type calcium current. Old pacemaker cells showed a reduction in L-type calcium current across all voltages tested, with no apparent shift in voltage dependence (Figure 3D and 3E). We next sought to investigate the underlying mechanism behind the reduction in the L-type calcium current.

### Aging Is Not Associated With Detectable Cell Hypertrophy

The effects of aging on the size of the heart are widely recognized. A well-accepted age-associated alteration is increased ventricular wall thickness,^26–28^ which is underlined by cardiomyocyte hypertrophy. Age-associated structural changes in the pacemaker have been previously reported, including loss of cell number^29^ and an increase in fibrosis.^30^ We tested whether cell hypertrophy could explain the reduced I_CaL_. To assess changes in pacemaker cell size, we used 3 different strategies. First, we quantified the cell size from high-resolution volumetric reconstructions of the sinoatrial node explants. Explants were isolated and immunostained against HCN4 (Figure S1A). Individual cells had an average width of 6.2±0.3 µm in young and 6.7±0.3 µm in old mice (Figure S1B), and an average length of 103±3 µm in young and 112±5 µm in old mice (Figure S1C). No statistically significant difference was observed in these morphological parameters. As a second approach, we measured the same morphological parameters from single, isolated pacemaker cells immunostained after tissue dissociation (Figure S1D through S1G). We found that neither the width (7.1±0.3 µm in young versus 8.4±0.3 µm in old), length (96±4 µm in young versus 99±3 µm in old), nor the area (627±36 μm^2^ in young versus 702±33 μm^2^ in old) was significantly different in isolated cells. As a last approach to evaluate changes in cell size, we quantified the cell capacitance. No statistically significant difference was observed in capacitance between young (26.8±2.0 pF) and old (27.2±1.8 pF) pacemaker cells (Figure S1H). Together, these analyses did not detect statistically significant age-associated differences in pacemaker cell size, suggesting that the reduction in L-type calcium current density is not accompanied by detectable cellular hypertrophy.

### Reduction in L-Type Calcium Current in Old Pacemaker Cells Is Not Associated With Detectable Changes in the Calcium Clearance Mechanism

Knockdown of the sodium-calcium exchanger 1 (NCX1) reduces I_CaL_,^31,32^ so we asked whether aging alters NCX1 expression or activity. We assessed total NCX1 expression by Western blot. NCX1 was 2-fold more expressed in the old pacemaker tissue compared with the young (Figure S2A). To determine the changes in NCX1 expression specifically in pacemaker cells, we performed immunocytochemistry in isolated cells from young and old animals against HCN4 and NCX1 (Figure S2B). Airyscan imaging was used to calculate the total area occupied by NCX1 (Figure S2C) and the NCX1 density per unit area (Figure S2D). Both measurements confirmed that old pacemaker cells have a significantly higher expression of NCX1. We noticed that a high proportion of the NCX1 signal localized intracellularly. Using the HCN4 membrane labeling as a mask, we quantified the percentage of NCX1 localized at the plasma membrane relative to that localized in intracellular membranes and found no significant difference between age groups, with a percentage at the plasma membrane of 34.9±5.1% in the young and 34.1±3.3% in the old (Figure S2E and S2F).

Aging has been previously associated with a decrease in sarcoplasmic reticulum (SR) calcium load.^33^ To determine whether the decreased L-type calcium current in old pacemaker cells is also accompanied by a change in the SR calcium load, we performed caffeine-induced SR calcium release experiments. We measured SR calcium load using caffeine-induced calcium release in Cal-520-loaded cells (Figure S2G). In agreement with Liu et al, we found that old pacemaker cells exhibited a 20% reduction in SR load, with a peak amplitude mean fold change of 3.3±0.2 compared with 4.1±0.2 in young cells (Figure S2H). Since NCX1 is the main calcium extrusion pathway in pacemaker cells, we used the decay time of the caffeine-induced transient as a proxy for NCX1 activity. The decay time in young cells was 1.21±0.14 seconds and in old cells was 1.19±0.13 seconds (Figure S2I), indicating no significant change in calcium extrusion despite the increase in NCX1 expression, which may only be a compensatory mechanism. Together, these analyses fail to provide evidence that impairments in calcium clearance mechanisms contribute to the age-associated reduction in L-type calcium current.

### Reduction in L-Type Calcium Current in Old Pacemaker Cells Is Associated With a Decrease in Surface Expression and Clustering at the Plasma Membrane

**Figure 4.**
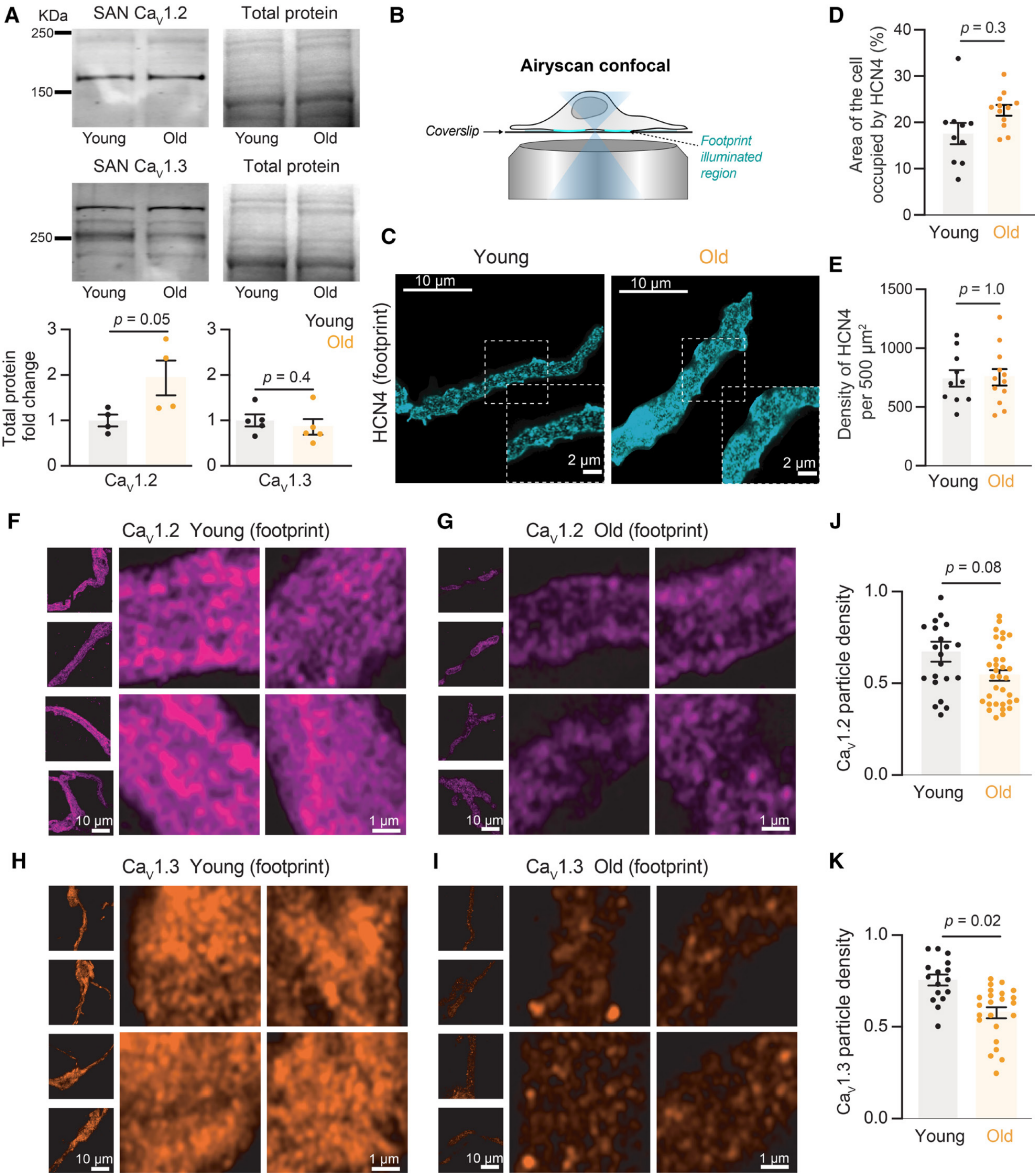
**Aging does not change total L-type calcium channel expression but reduces their density near the plasma membrane. A**, Representative Western blots for L-type voltage-gated calcium channel α1C subunit (Ca_V_1.2) and L-type voltage-gated calcium channel α1D subunit (Ca_V_1.3) with total protein stain from pacemaker tissue explants from young and old mice. Fold change of total Ca_V_1.2 and Ca_V_1.3 expression in old vs young, normalized to total protein; each point represents 1 animal (Ca_V_1.2 N=4 young, 4 old; Ca_V_1.3 N=5 young, 5 old). **B**, Diagram of cell-footprint imaging by Airyscan confocal microscopy. **C**, Representative Airyscan images of the footprint from young and old cells labeled against hyperpolarization-activated cyclic nucleotide-gated channel isoform 4 (HCN4; cyan); insets, magnified regions. **D**, Percentage of cell area occupied by HCN4 signal, young vs old. **E**, HCN4 particle density, young vs old (D, E young n=10 cells from 3 mice; old n=12 cells from 3 mice). **F** through **I,** Representative Airyscan footprint images from young and old cells labeled against Ca_V_1.2 (magenta) or Ca_V_1.3 (orange); insets are 5×5 µm regions of each cell. **J** and **K**, Ca_V_1.2 and Ca_V_1.3 particle density, young vs old (Ca_V_1.2 young n=22 cells from 3 mice, old n=33 from 3; Ca_V_1.3 young n=16 from 3, old n=23 from 3). Bars are mean±SEM. Statistical comparisons in **A** used 2 independent Mann-Whitney *U* tests and in panels **D**, **E**, **J**, **K** nested *t* tests to account for cells nested within animals.

First, we evaluated whether the observed age-associated reduction in current density was caused by a reduction in the abundance of L-type calcium channels. We measured the Ca_V_1.2 and Ca_V_1.3 protein levels by Western blot from protein lysates obtained from sinoatrial node explants from young and old animals. Contrary to our prediction, total protein levels were not decreased in samples from old animals. Instead, Ca_V_1.2 channels were 1.9±0.4-fold higher in old animals compared with the young. The abundance of Ca_V_1.3 channels was the same in old and young animals (0.9±0.2-fold relative to the young; Figure 4A).

As total channel expression was not altered, we next assessed surface expression using Airyscan microscopy to image the cell footprint, which is the region of the cell in close contact with the glass coverslip (Figure 4B). Figure S3 shows in detail the description and validation of the Airyscan footprint using the membrane marker wheat germ agglutinin and comparing to total internal reflection fluorescence microscopy. For assessing Ca_V_1.2 or Ca_V_1.3 expression, we colabeled with HCN4. HCN4 is a robust membrane marker (Figure S1D) that exhibits a clear footprint (Figure 4C) while allowing us to identify pacemaker cells. We quantified the intensity of HCN4 at the footprint as a control and found no significant differences between young and old pacemaker cells (Figure 4D and 4E). The footprint Z plane identified in the HCN4 channel was used to acquire the images for Ca_V_1.2 or Ca_V_1.3. Ca_V_1.2 and Ca_V_1.3 labeling at the footprint was nonhomogeneous and dotted. Old cells showed 19% fewer Ca_V_1.2 particles per unit membrane area, with a density of 0.54±0.03 particles/μm^2^, compared with 0.67±0.05 particles/μm^2^ in young cells (Figure 4F, 4G, and 4J). Ca_V_1.3 particle density was reduced in old pacemaker cells by 24%, with values of 0.75±0.03 particles/μm^2^ for the young and 0.58±0.03 particles/μm^2^ for the old (Figure 4H, 4I, and 4K).

**Figure 5.**
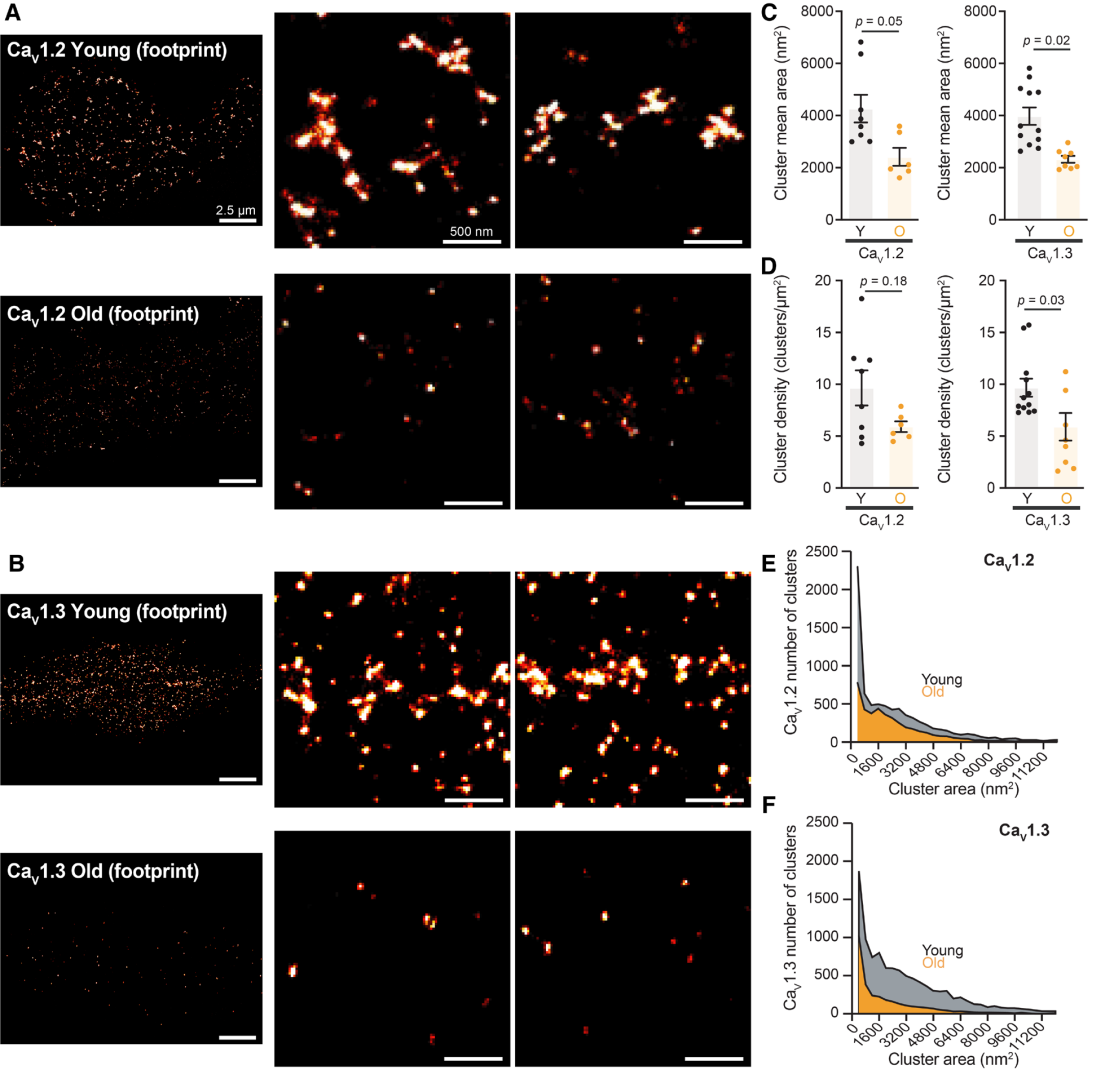
**Aging reduces L-type calcium channel clustering. A** and **B**, Representative super-resolution images of L-type voltage-gated calcium channel α1C subunit (Ca_V_1.2) and L-type voltage-gated calcium channel α1D subunit (Ca_V_1.3) channels in young and old pacemaker cells at the footprint plane. Right panels: magnification. **C**, Average cluster area for Ca_V_1.2 and Ca_V_1.3 in young vs old cells. **D**, Ca_V_1.2 and Ca_V_1.3 cluster density, young vs old. **E** and **F**, Frequency distributions of Ca_V_1.2 and Ca_V_1.3 cluster areas, young vs old. Bars are mean±SEM (Ca_V_1.2 young n=8 cells from 3 mice, old n=6 from 3; Ca_V_1.3 young n=12 from 4, old n=8 from 3). Statistical comparisons used independent nested *t* tests for each channel.

As a second approach, we compared the organization of L-type calcium channels at a nanometer resolution. Using stochastic optical reconstruction microscopy in total internal reflection fluorescence mode, we quantified channel cluster density at ≈20-nm resolution. In agreement with the previous experiment, old cells exhibited less channels per membrane unit area. For the Ca_V_1.2 channels, cluster density was 9.6±1.7 clusters per μm^2^ in young and 5.9±0.5 clusters per μm^2^ in old cells; although the values trended lower in old cells, no statistically significant difference was observed (Figure 5A, 5B, and 5D). In contrast, Ca_V_1.3 cluster density was significantly reduced by 39%, going from 9.7±0.9 clusters per μm^2^ in young to 5.9±1.3 clusters per μm^2^ in old cells (Figure 5D). Consistent with prior work showing L-type channels organize in clusters,^34–37^ young cells exhibited cluster sizes of 4265±531 nm^2^ for the Ca_V_1.2 and 3975±334 nm^2^ for the Ca_V_1.3 (Figure 5A through 5C). Cells from old animals showed a significant reduction in Ca_V_1.3 mean cluster area, with values of 2321±132 nm^2^ in old compared with 3975±334 nm^2^ in young (Figure 5A through 5C). Ca_V_1.2 cluster area trended smaller in old cells (2416±342 nm^2^ versus 4265±531 nm^2^ in young; Figure 5A through 5C), but this difference did not reach statistical significance (*p*=0.054). Figure 5E and F show the frequency distribution for the Ca_V_1.2 and Ca_V_1.3 channel cluster area. The cluster area frequency distribution was significantly shifted down and to the left in old cells for Ca_V_1.3, with a nonsignificant trend in the same direction for Ca_V_1.2 (Figure 5E and 5F), suggesting an age-associated reduction in L-type channel cluster size that is most pronounced for Ca_V_1.3. Taking together the results from these 2 approaches, we concluded that aging reduces the number of L-type calcium channels inserted at the plasma membrane but not their total expression, and that this reduction in density at the plasma membrane leads to a reduction in the number of channels found in clusters.

### Reduction in L-Type Calcium Current in Old Pacemaker Cells Is Associated With a Decrease in the Activity of Single Channels

**Figure 6.**
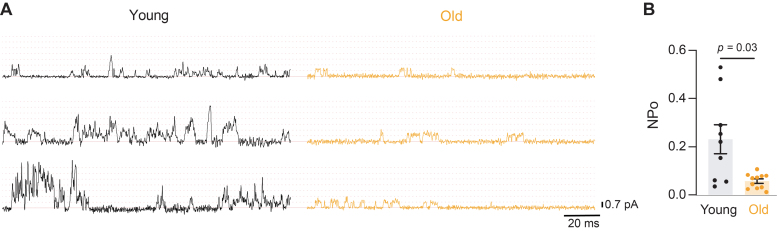
**Aging reduces the number of active channels at the plasma membrane and their open probability. A**, Representative single-channel recordings from young (black) and old (orange) pacemaker cells. Dotted lines mark openings of 1 to 7 channels from the closed state (unitary current 0.7 pA). **B**, Channel open probability (NP_o_), young vs old, calculated from 50 voltage sweeps per cell. Statistical comparisons used a nested *t* test (young n=9 cells from 3 mice; old n=11 cells from 3 mice).

To further test the hypothesis that aging leads to a reduction of channels at the plasma membrane and that fewer channels result in a reduction in L-type calcium current, we assessed the activity of single channels in small membrane patches. Figure 6A shows representative single-channel recordings from young and old pacemaker cells. The channel activity in patches from young cells was characterized by coordinated opening of multiple channels. Notably, single-channel recordings in old cells were dominated by independent single-channel events that rarely exhibited coordinated opening of multiple channels. We calculated number of channels times open probability for each patch. This parameter was around 4× larger in young (0.23±0.06) than old (0.06±0.01) patches (Figure 6B). The change in channel open probability measured by patch electrophysiology was considerably larger than the change in channel density measured by microscopy, suggesting that aging not only affects the number of channels but also their open probability.

### Increasing L-type Calcium Channel Open Probability Restores Firing Rates in Pacemaker Cells From Old Animals

**Figure 7.**
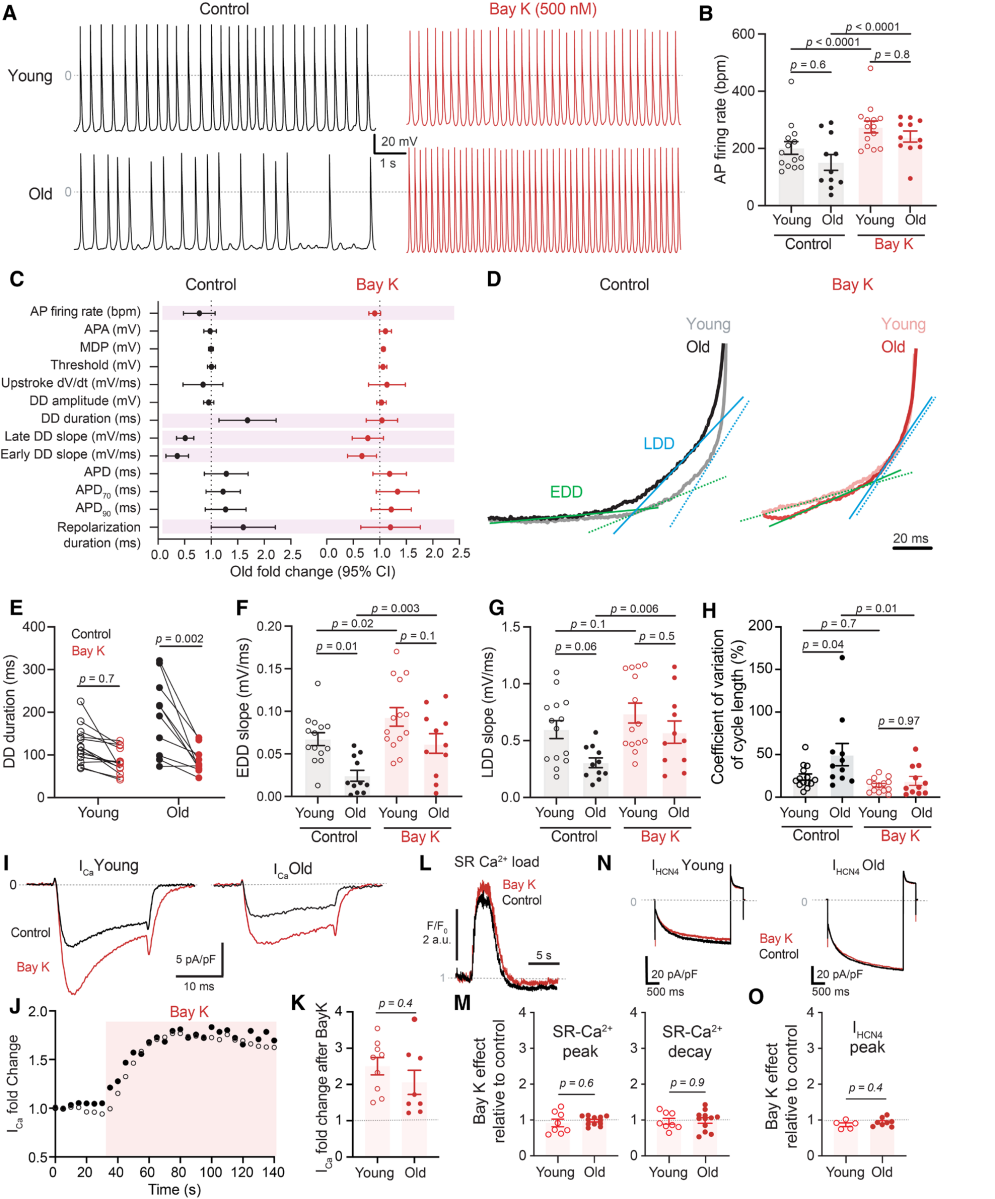
**Increasing L-type calcium channel open probability restores normal pacemaking rates in old cells. A**, Representative action potentials from young and old cells before (control, black) and after (red) application of 500 nmol/L Bay K 8644 (Bay K). **B**, Effect of Bay K on firing rate, young vs old. **C**, Fold change of action potential parameters in old vs young, under control (black) and Bay K (red). Vertical dotted lines represent the young population; purple bars indicate parameters significantly different under control but reverted by Bay K. **D**, Representative overlapped magnification of the diastolic depolarization under control and Bay K. Straight lines indicate early diastolic depolarization (EDD; green) and late diastolic depolarization (LDD; blue) slopes from young (dotted) and old (solid) traces. **E**, Effect of Bay K (red) on diastolic depolarization (DD) duration relative to control (black), young vs old. **F** and **G**, EDD (**F**) and LDD (**G**) slopes, young vs old, under control (black) and Bay K (red). **H**, Coefficient of variation (CV) of cycle length, young vs old, under control (black) and Bay K (red). **I**, Representative calcium current recordings under control and Bay K, young and old. **J**, Representative time courses of the Bay K effect on calcium currents. **K**, Bay K effect on calcium currents as fold change relative to control (young n=9 cells from 3 mice; old n=8 from 3). **L**, Representative caffeine-induced calcium responses (SR Ca^2+^ load) under control and Bay K in old cells. **M**, Bay K effect on peak and decay of the caffeine-induced calcium transient (young n=8 from 3; old n=12 from 3). **N** Representative I_HCN4_ recordings under control and Bay K, young and old. **O**, Bay K effect on I_HCN4_ amplitude (young n=5 from 2; old n=8 from 3). Bars are mean±SEM. **E**, 2 paired 2-tailed *t* tests within each age group (young n=14 cells from 3 mice; old n=10 from 3). Statistical comparisons for panels **B**, **F**, **G**, **H** used a linear mixed effects model (lme4 in R) with Sidak correction over 4 prespecified contrasts (young control vs young Bay K; old control vs old Bay K; young vs old under control; young vs old under Bay K); young n=14 from 3; old n=11 from 3. For panels **K**, **M**, **O** nested *t* tests were used.

If a decrease in the open probability of L-type calcium channels is one of the factors by which aging slows down the pacemaker rate, one would predict that increasing it in old pacemaker cells would accelerate their firing rate. To test this hypothesis, we recorded the spontaneous firing rate of isolated young and old pacemaker cells in control conditions and under the bath application of 500 nmol/L of the L-type channel agonist Bay K 8644 (Bay K). Bay K has been shown to increase the open probability and opening time of L-type calcium channels.^38–43^ Under control conditions, firing rate trended slower in old cells, consistent with Figure 2 (Figure 7A, left). The perfusion of Bay K accelerated the spontaneous firing rate in young cells 1.3-fold, going from 201±22 bpm to 276±20 bpm (Figure 7B). The effect of Bay K in old cells was stronger, with an acceleration of 1.6-fold, going from 151±27 to 242±19 bpm (Figure 7A and 7B). Remarkably, increasing L-type calcium channel open probability with Bay K was enough to accelerate the firing rate in old cells to values not statistically different from the young group (Figure 7B). Increasing the open probability of L-type calcium channels had a similar effect on other action potential parameters. The application of Bay K reverted the significant differences observed in control conditions between young and old for the DD duration, EDD and LDD slopes (Figure 7C and 7D). A paired analysis of the DD showed that Bay K significantly shortened DD only in old cells (Figure 7E). Bay K significantly increased EDD slope in both groups (Figure 7F and 7G), and significantly increased LDD slope in old cells while producing a nonsignificant trend in young cells. In control conditions, EDD was significantly slower in old cells (0.02±0.01 versus 0.07±0.01 mV/ms) and LDD trended in the same direction (0.30±0.04 versus 0.60±0.08 mV/ms), consistent with Figure 2 (Figures 7F, 7G). Under Bay K application, EDD and LDD were significantly accelerated in the old group (EDD: 0.09±0.01 mV/ms in young versus 0.06±0.01 mV/ms in old; LDD: 0.74±0.09 mV/ms in young versus 0.58±0.01 mV/ms in old cells). EDD and LDD were accelerated 1.4 and 1.25×, respectively, in young cells. In contrast, Bay K accelerated EDD 2.6× and LDD 1.9 times in old cells. Bay K also abrogated beat-to-beat variability. SD of cycle length in old cells dropped from 309±112 ms to 52±20 ms with Bay K, becoming statistically indistinguishable from young (97±22 ms control; 36±6 ms with Bay K). The coefficient of variation of cycle length in old cells dropped from 50.0±13.1% under control to 19.1±5.2% with Bay K, becoming statistically indistinguishable from young (23.6±3.6% control; 14.1±2.1% with Bay K, Figure 7H). Together, these results suggest that increasing the open probability of L-type calcium channels in old pacemaker cells contributes to pacemaker rate acceleration through an increase in the DD rate.

We next tested the specificity of Bay K. Figure 7I through 7K shows that the application of 500 nmol/L Bay K led to comparable fold-changes in calcium currents from young and old cells, with no statistically significant difference between groups (2.5 ± 0.2-fold in young and 2.1 ± 0.3-fold in old). In contrast, Bay K had no statistically significant effect on the amplitude of the calcium response to caffeine (fold change 0.92±0.1 in young and 0.97±0.03 in old) or the decay time (fold change 0.97±0.08 in young and 0.99±0.08 in old; Figure 7L and 7M). No statistically significant change in the amplitude of the HCN current was observed (fold change 0.87±0.05 in young and 0.94±0.04 in old; Figure 7N and 7O). Together, these results suggest that Bay K increases the firing rate of old pacemaker cells by an increase in the open probability of the fraction of L-type calcium channels that remain in the plasma membrane of old pacemaker cells. Our results also support the claim that a reduction in the open probability of L-type calcium channels is one of the mechanisms by which aging slows down the firing frequency of pacemaker cells.

### Link Between Aging and Reduced L-Type Calcium Channel Surface Expression Is the Loss of Caveolin-3

What connects the process of aging to the reduction of L-type calcium channel surface expression? To address this question, we tested the hypothesis that aging disrupts caveolae and, therefore, reduces L-type calcium channel density. Unlike working cardiomyocytes that possess T-tubules, pacemaker cells rely on an extensive caveolar network where L-type calcium channels and other channels and receptors reside. Notably, aging has been associated with caveolar disruption in ventricular cardiomyocytes and other cell types.^44–47^ Supporting this, pacemaker cells from caveolin-3 knockout mice exhibit reduced L-type calcium currents,^48^ and our prior work demonstrated that aging diminishes the interaction between Ca_V_1.2/Ca_V_1.3 channels and caveolin-3.^49^

**Figure 8.**
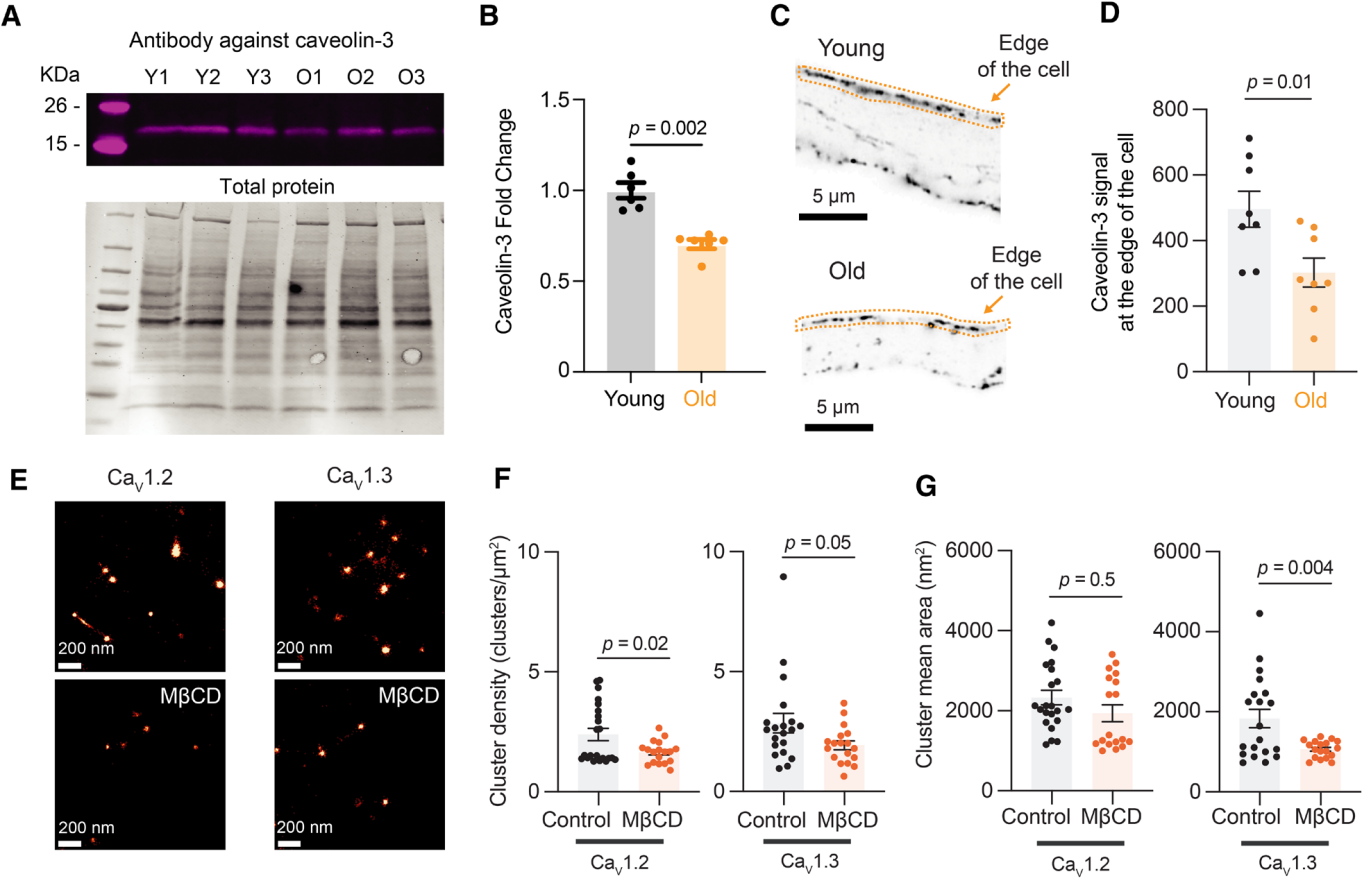
**The link between aging and reduced L-type calcium channel surface expression is the loss of caveolin-3. A**, Representative immunoblot of caveolin-3 with total protein stain from pacemaker tissue explants from young and old mice. **B**, Fold change of caveolin-3 expression in old vs young, normalized to total protein; each point represents 1 animal (N=6 young, 6 old). **C**, Representative Airyscan images of young and old cells labeled against caveolin-3. Orange dotted line marks the membrane region where signal was measured. **D**, Caveolin-3 signal intensity, young vs old (young n=8 cells from 3 mice; old n=8 from 3). **E**, Representative super-resolution images of L-type voltage-gated calcium channel α1C subunit (Ca_V_1.2) and L-type voltage-gated calcium channel α1D subunit (Ca_V_1.3) channels in young cells with or without methyl-β-cyclodextrin (MβCD). **F**, Ca_V_1.2 and Ca_V_1.3 cluster density, MβCD vs control. **G**, Mean cluster area for Ca_V_1.2 and Ca_V_1.3, MβCD vs control (**F** and **G**: Ca_V_1.2 control n=22 cells from 3 mice, MβCD n=18 from 3; Ca_V_1.3 control n=20 from 3, MβCD n=18 from 3). Bars are mean±SEM. Statistical comparisons use a 2-tailed Mann-Whitney *U* test for **B** and nested *t* tests for panels **D**, **F**, **G**.

To test this hypothesis, we first quantified caveolin-3 abundance in young and old pacemaker cells using Western blotting and immunocytochemistry. Caveolin-3 levels were reduced by 30% in old animals (Figure 8A and 8B), as measured by Western blot. Consistent with this, caveolin-3 immunofluorescence intensity in isolated old pacemaker cells was reduced by 40% (Figure 8C and 8D). We next assessed whether disrupting the caveolar network alters L-type calcium channel surface expression. Young cells were treated with 5 mmol/L methyl-β-cyclodextrin (MβCD) for 30 minutes to disrupt caveolae.^50^ Super-resolution imaging revealed that MβCD treatment reduced Ca_V_1.2 cluster density by 30% (2.4±0.3 clusters per μm^2^ in control versus 1.6±0.1 clusters per μm^2^ in MβCD; Figure 8E and 8F). Ca_V_1.3 cluster density was also reduced by 30% in MβCD-treated cells (2.8±0.4 clusters per μm^2^ in control versus 1.9±0.2 clusters per μm^2^ in MβCD; Figure 8E and 8F).

In addition to reduced surface expression, aging was also associated with smaller L-type calcium channel clusters (Figure 5). We therefore tested whether caveolar disruption affects cluster size. MβCD treatment did not reduce Ca_V_1.2 cluster size but decreased Ca_V_1.3 cluster size by 40%, from 1832±232 nm^2^ in control to 1063±47 nm^2^ in MβCD (Figure 8G). Together, these experiments support the hypothesis that age-associated loss of caveolin-3 in pacemaker cells contributes to the reduction in L-type calcium channel surface expression.

## Discussion

Our study aimed to elucidate the molecular mechanisms underlying the age-related slowdown of the cardiac pacemaker. We provide evidence that the L-type calcium current is reduced in aged pacemaker cells and identify specific changes in L-type calcium channels: (1) decreased channel density at the plasma membrane, (2) smaller channel clusters, and (3) reduced open probability. These findings strengthen the concept that impaired calcium signaling contributes to pacemaker dysfunction with aging. We also identified the loss of caveolin-3 as a potential mechanism linking aging with the reduced expression of L-type calcium channels.

Importantly, our data allow us to exclude several alternative explanations, including changes in cell size, compensatory upregulation of other proteins, and alterations in voltage dependence. Limitations of our study include the use of male mice only, with sex-based differences remaining to be investigated, and the unresolved molecular events linking aging to reduced caveolin-3 expression.

### Cell Size and Previous Reports

We examined whether pacemaker cells undergo hypertrophy with aging. Contrary to 2 previous reports, our results indicate that cell size does not increase in old mice. The discrepancy may reflect differences in species, age ranges, or measurement techniques. One study reported a 33% increase in cell size in 28-month-old mice compared with 2-month-old mice.^1^ Measurements were taken with electrophysiological equipment and reported as cell capacitance. This is considered a highly sensitive technique, as it can detect minute changes in cell capacitance (cell size) as little as the addition of membrane from a single vesicle, which would be impossible to detect using microscopy. One limitation of this approach is that the cell size and shape are expected to change as the recording solution from the pipette dialyzes into the cell due to differences in osmolarity. Another study in rats found a 38% increase in cell diameter using histological sections. This study compared 3-month-old and 24-month-old rats.^51^ We used 3 complementary approaches to address this discrepancy: tissue clearing to measure cell dimensions in situ, high-resolution imaging of isolated cells, and capacitance recordings. Across all 3 methods, no statistically significant change in pacemaker cell size was detected with aging.

### Role of Age-Associated Loss of Caveolar Microdomains

The caveolar network serves as specialized signaling microdomains. Interestingly, aging has been linked to caveolar disruption in multiple tissues, including the heart.^44–47^ This age-associated caveolar disruption has been associated with changes in autophagy,^52^ reduced endocytosis,^53^ and cellular disorganization.^54^ Given the caveolar network’s role in maintaining the proper organization of signaling molecules in pacemaker cells, it is possible that caveolar loss contributes to age-associated dysfunction of this tissue. Our group recently demonstrated that aging is associated with disrupted caveolar domains and reduced interaction between Ca_V_1.2/Ca_V_1.3 and caveolin-3.^49^ Now, we added more evidence supporting this model. We show that aging reduces caveolin-3 expression, which could explain the disrupted caveolar network and reduced interactions with Ca_V_1.2 and Ca_V_1.3 channels. Our results also show a causal relationship between caveolar loss and reduced surface expression of Ca_V_1.2 and Ca_V_1.3 channels. These results are in line with functional studies showing that the loss of caveolin-3 reduces calcium currents.^48,55,56^

### Alternative Mechanisms Linking Aging to Reduced L-Type Channel Surface Expression

Another alternative explanation for the reduced surface expression is an age-associated loss of proteostasis, a well-recognized hallmark of aging.^57,58^ Misfolding or translational errors could lead to channel degradation or trafficking defects, preventing proper membrane insertion. Loss of proteostasis in the heart is strongly associated with cardiac dysfunction.^59^ Furthermore, aging hearts exhibit increased protein oxidation^59,60^ and ubiquitination,^61^ both characteristic of proteostasis decline. Proteomic remodeling during aging further supports this concept: proteins involved in oxidative metabolism (electron transport chain, citric acid cycle, and fatty acid metabolism) decrease, whereas glycolytic enzymes and extracellular matrix proteins increase.^62^ These changes suggest aging alters ionic currents through impaired trafficking and degradation. Whether this effect is specific to L-type calcium channels remains unclear. Our data show that NCX1 and HCN4 surface expression is preserved, but other channels and receptors should be examined.

Another potential mechanism relates to scaffolding proteins. Known scaffolds for L-type calcium channels include BIN1,^63,64^ AKAPs,^65–69^ and Shank.^70–72^ BIN1 facilitates Ca_V_1.2 delivery and clustering in ventricular myocytes, whereas AKAP150 supports cooperative gating in smooth muscle cells. However, the function of the majority of these scaffolds remains to be tested in pacemaker cells. Phosphorylation also regulates clustering. For example, phosphorylation at S1928 promotes Ca_V_1.2 superclustering in smooth muscle cells,^73^ and phosphorylation at S1700 mediates clustering in ventricular myocytes via 14-3-3 interaction.^74^ We speculate that smaller channel clusters in aged cells may also result from altered scaffolding proteins. Future studies should identify key phosphorylation sites in the channels expressed in pacemaker cells and determine whether aging alters these modifications.

### Reduced Open Probability and Functional Coupling

Our results show that L-type calcium channels exhibit reduced open probability in aged pacemaker cells. Notably, increasing the open probability of L-type calcium channels with Bay K restored firing frequency in old cells to young levels, supporting our conclusion that the reduction in the open probability of L-type calcium channels is a key contributor to the age-associated slowdown. What underlies this reduction?

L-type channels operate in 2 gating modes^40^: Mode 1 (short openings, low open probability) and Mode 2 (long openings, high open probability). Bay K increases Mode 2 prevalence, both in single-channel recordings and calcium sparklets.^75^ Mode 2 activity depends on channel clustering, which enables C-terminal interactions mediated by calmodulin—a mechanism we previously described for Ca_V_1.2 and Ca_V_1.3.^34,35^ Aging may reduce open probability by impairing this coupling through smaller clusters and fewer adjacent channels. Our findings support this hypothesis, but direct characterization of channel coupling in aged pacemaker cells is needed.

An alternative explanation for our findings involves the role of Ca_V_1.3 channels in coupling the membrane and calcium clocks in pacemaker cells. The membrane clock depends on the activation of ion channels such as HCN4 and Ca_V_1.3, whereas the calcium clock relies on local calcium release events mediated by ryanodine receptors and subsequent NCX activation. Ca_V_1.3 channels have been proposed to facilitate the coupling between these 2 mechanisms. We speculate that Bay K restores pacemaker function by enhancing coupling between the membrane and calcium clocks. Three lines of evidence support this hypothesis: (1) Ca_V_1.3 knockout mice exhibit reduced local calcium release events.^76^ (2) Pacemaker cells from old mice exhibit reduced local calcium release events.^33^ (3) Bay K application increases local calcium release events in young pacemaker cells.^77^ The fact that Bay K abrogates beat-to-beat variability, which depends on the calcium clock, further supports this hypothesis. Future studies should assess the level of coupling between the membrane and calcium clocks in aged pacemaker cells.

## Conclusions

The present findings have important implications for understanding cardiac aging. Pacemaker dysfunction is a major contributor to bradyarrhythmias and the increased need for pacemaker implantation in older adults. By identifying functional and structural alterations in L-type calcium channels, our work shifts the focus toward channel gating and organization as key mechanisms driving pacemaker failure. Interventions aimed at preserving channel organization, enhancing clock coupling, or restoring open probability could offer novel strategies to maintain cardiac rhythm in older adults.

## Article Information

### Acknowledgments

The authors thank Michael Lai for technical support.

### Disclosures

None.

### Supplemental Material

Expanded Materials and Methods

Figures S1–S3

Uncropped Western blots

Major Resources Table

References 37,66,68,78,79

## Supplementary Material
