# Supplementary material for: Aging Disrupts L-type Ca2+ Channel Organization and Function in Pacemaker Cells
Source: Circ Res. 2026 Jun 19;139(4):e327894. doi: 10.1161/CIRCRESAHA.125.327894 (PMC13418876; doi:10.1161/CIRCRESAHA.125.327894)
Supplement: Supplementary file 1 [file res-139-e327894-s001.pdf]

SUPPLEMENTAL MATERIAL

Aging Disrupts L-type Ca^{2+} Channel Organization and Function in Pacemaker Cells

Oscar Vivas[1,2]*, Matthias Baudot[2]*, Roxanne Madden[2], Wendy L. Piñon-Teal[2], Martina S. Hunt[2], Sabrina Choi[2], Roya Pournejati[1], Victor A. Flores-Tamez[3], L. Fernando Santana[4], Claudia M. Moreno[2,5 †]

Affiliations:

1 Department of Pharmacology, University of Washington, Seattle, WA 98195, USA

2 Department of Neurobiology and Biophysics, University of Washington, Seattle, WA 98195, USA

3 Department of Pharmacology, University of California, Davis, CA 95616, USA

4 Department of Physiology and Membrane Biology, University of California, Davis, CA 95616, USA

5 Howard Hughes Medical Institute, University of Washington, Seattle, WA 98195, USA

*Vivas O and Baudot M are Co-first authors

† Corresponding author: Claudia M. Moreno

Email: morenocm@uw.edu

EXPANDED MATERIALS AND METHODS

All data and materials have been made publicly available at Dryad and can be accessed at https://doi.org/10.5061/dryad.sn02v6xmb.

Solutions

For the isolation of pacemaker cells, the following solutions were used: Tyrode's III solution (in mM) – 148 NaCl, 5.4 KCl, 5 HEPES, 5.5 glucose, 1 $MgCl_2$, and 1.8 $CaCl_2$, pH was adjusted to 7.4 with NaOH. Low-Calcium Tyrode's solution (in mM) – 140 NaCl, 5.4 KCl, 5 HEPES, 5.5 glucose, 1.2 KH_2PO_4, 50 taurine, 0.5 $MgCl_2$, and 0.2 $CaCl_2$, pH was adjusted to pH 6.9 with NaOH. KB solution (in mM) – 80 L-glutamic acid, 25 KCl, 10 HEPES, 10 glucose, 10 KH_2PO_4, 20 taurine, 0.5 EGTA, and 3 $MgCl_2$, pH was adjusted to 7.4 with KOH.

For electrophysiological recordings, the following solutions were used: Whole-cell voltage-clamp calcium current (I_{Ca}) external solution (in mM) – 110 N-methyl-D-glutamine, 5 CsCl, 10 HEPES, 10 glucose, 30 TEA-Cl, 4 4-Aminopyridine, 1 $MgCl_2$, and 2 $CaCl_2$, pH was adjusted to 7.4 with HCl. I_{Ca} internal solution (in mM) – 50 CsCl, 10 HEPES, 70 L-Aspartic acid, 30 TEA-Cl, 5 EGTA, 5 Mg-ATP, 1 $MgCl_2$, and 0.7 $CaCl_2$, pH was adjusted to 7.2 with CsOH. Perforated-patch current-clamp action potential external solution was Tyrode's III. Perforated-patch current-clamp action potential internal solution (in mM) – 130 L-Aspartic acid K, 10 NaCl, 10 HEPES, 0.04 $CaCl_2$, 2 Mg-ATP, 0.1 Na-GTP, 6.6 phosphocreatine, pH was adjusted to 7.2 with KOH. Cell-attached single channel recording external solution (in mM) – 145 KCl, 10 NaCl, 10 HEPES, pH was adjusted to 7.4 with NaOH. Cell-attached single channel recording internal solution (in mM) – 110 $CaCl_2$, 20 TEA-Cl, 10 HEPES, and 10 μM of the HCN channel-blocker ivabradine, pH was adjusted to 7.2 with CsOH.

For protein extraction, the following solutions were used: Homogenization buffer (in mM) – 150 NaCl, 50 Trizma base (from 1M stock at pH 7.8 with HCl), 5 EDTA (from 50 mM stock pH 7.8 with NaOH); the day before the experiment NaF 10 mM, Glycerol 5% v/v, Triton X-100 1% v/v, Sodium deoxycholate 0.25% were added; on the day of the

experiment one tablet of SIGMAFAST^{TM} protease inhibitor cocktail (Sigma, S8830), EDTA free in 50 ml of the buffer was added. Lysis Buffer (to make 10 ml) – 10 ml of homogenization buffer, 8.3 µl Calpain I (10 mg/ml) (Fisher, AAJ61766LB0), 8.3 µl Calpain II (10 mg/ml) (Sigma, A6060), 10 µl o-phenathroline (200 mg/ml) (Sigma, 33510), 10 µl PMSF (20 mg/ml) (Sigma, P7626), 10 µl Benzamidine (15.7 mg/ml) (Sigma, 12072), prepared the day of the experiment. Solutions were kept on ice.

Pacemaker whole-mount immunohistochemistry and clearing

The SAN explant was dissected using anatomical landmarks as in Figure 1 and fixed in 4% paraformaldehyde in PBS for 30 min at room temperature. After fixation, SAN preparations were rinsed in PBS and blocked in PBS with 0.25% Triton X-100 and 3% BSA. Tissue was incubated with rabbit polyclonal anti-HCN4 at 1:250 (Millipore Sigma, AB5808) for 48 h at 4°C with constant rocking. Secondary antibodies conjugated to Alexa 555 fluorophores were incubated at 1:500 in blocking solution overnight at room temperature. Clearing was performed by graded dehydration in ethanol followed by immersion in ethyl cinnamate (ECi). SANs were transferred sequentially into 25%, 50%, 75%, and 100% ethanol (5 min each), then into a second 100% ethanol tube for 5 min. Tissues were then placed in 100% ECi for 3 min, followed by a second ECi bath for ≥10 min before mounting. Cleared SANs were imaged using a Zeiss AiryScan microscope.

Pacemaker cells isolation

Pacemaker cells were freshly isolated from mature adults (4-6 months old), or old (24-30 months old) C57BL/6 male mice. Mice were housed in an AAALAC-accredited, pathogen-free animal facility under standard housing conditions, including a 12 h light/dark cycle and ad libitum access to standard laboratory chow and water. All work with animals was performed under an approved protocol by the University of Washington Institutional Animal Care and Use Committee (IACUC). Animals were euthanized by intraperitoneal overdose of Euthasol (Virbac, 400mg/ml). The heart was dissected and pinned down on a Sylgard-coated dish containing warm Tyrode's III solution. The sinoatrial node region was identified under the stereoscope as the region delimited at the top by the superior vena cava, at the right by the sulcus terminalis, and the bottom by the coronary sulcus

and the inferior vena cava (Figure 1A). The sinoatrial node artery was also used as an anatomical landmark to identify the pacemaker region. Pacemaker cells were then isolated from the sinoatrial node following the protocol described by Fenske et al. 2016[78]. Briefly, the excised sinoatrial node tissue was immersed in a 37°C pre-heated 2 ml tube containing 675 µl of Low-Calcium Tyrode's. After 5 min of stabilization at 37°C the following compounds were added to the tube: BSA (Cf = 1 mg/ml), elastase (Cf = 18.87 U/ml, Millipore 324682), protease (Cf = 1.79 U/ml, Sigma P5147), and collagenase B (Cf = 0.54 U/ml, Roche 11088807001). The enzymatic digestion of the tissue was carried out for 30-35 minutes in a 37°C water bath, and a mechanical dissociation with a short fire-polished glass pipette was performed every 7 minutes during incubation. To stop the digestion, the digested tissue was centrifuged at 200 × g for 2 min at 4°C, and the supernatant was discarded and replaced with 1 ml of Tyrode's Low-Ca^{2+} solution; this process was repeated twice. Then the tissue was washed two times more with 1 ml of Calcium-free KB solution. The dissociated tissue was left to rest in KB solution at 4°C for a minimum of 40 min. Finally, single pacemaker cells were resuspended by applying gentle mechanical dissociation with a flame-forged glass pipette. For electrophysiology experiments, to recover the automaticity of the pacemaker cells, calcium was reintroduced into the KB cell's storage solution by the gradual addition of small amounts of Tyrode's III solution (10, 50 and 100 µl at 5 min intervals). Cells were plated on poly-L-Lysine (PLL)-coated coverslips. PLL coating was performed at least one day before the cell isolation. Clean coverslips were incubated in PLL hydrobromide solution MW 300,000 (Sigma, P1524) for 30 min at 37°C. Each coverslip was then removed onto a rack and flushed with Milli-Q water and aspirated 5 times. Coverslips were left to dry in a biosafety cabinet overnight.

Protein extraction and western blot

Animals were anesthetized, and the heart was quickly removed and placed in ice-cold dissection buffer. The heart was pinned on a Sylgard-coated 60 mm dish containing ice-cold dissection buffer, and the sinoatrial node explant was dissected as described above and quickly transferred into a glass micro-tissue grinder (DWK, 357844) containing 100 µl of solubilization buffer. The homogenization buffer base contained 50 mM Tris-HCl pH

7.8 (Sigma, T6066), 5 mM EDTA, 150 mM NaCl, 1% Triton X-100, 10 mM NaF, 0.25% sodium deoxycholate, and 5% glycerol. Dissection buffer was prepared fresh by supplementing 50 ml of the homogenization base with one SIGMAFAST™ Protease Inhibitor Cocktail Tablet, EDTA-free (Sigma, S8830). Solubilization buffer was prepared fresh by supplementing 10 ml of dissection buffer with calpain I (Fisher, AAJ61766LB0; final 8.3 μg/ml), calpain II (Sigma, A6060; final 8.3 μg/ml), o-phenanthroline (Sigma, 33510; final 0.2 mg/ml), PMSF (Sigma, P7626; final 20 μg/ml), and benzamidine (Sigma, 12072; final 15.7 μg/ml). The tissue was homogenized on ice, the sample was transferred to a 1.5 ml tube and placed on a rotating wheel at 4°C for 2 h. Samples were centrifuged at 16,000 × g for 20 min at 4°C, and the solubilized protein supernatant was transferred to a new tube and stored at −80°C. Protein concentration was determined using a Pierce BCA Protein Assay Kit (Thermo, 23225) following the manufacturer's instructions. For electrophoresis, 25 μg of protein were denatured in SDS loading buffer for 10 min at 70°C and loaded per lane on a Mini-Protean TGX Precast 4–15% Bio-Rad gel. Spectra Multicolor ladder (Thermo, 26634) was loaded as a molecular weight reference. Gels were run for 15 min at 50 V, followed by 1 h at 130 V in TGS.

For $Ca_V1.2$ and $Ca_V1.3$, proteins were transferred onto 0.2 μm PVDF membranes (Thermo, 1704157) using an Owl™ Electroblotting system (Thermo, VEP-2) at 0.4 A and 4°C for 2 h. Membranes were stained for total protein using No-Stain™ protein labeling reagent (Invitrogen, A44449) following the manufacturer's instructions, and imaged on an iBright imaging system (Thermo). For immunoblotting, membranes were blocked in 5% milk in TBST at RT for 1 h. Rabbit polyclonal anti-$Ca_V1.2$ (anti-CNC1) and rabbit polyclonal anti-$Ca_V1.3$ (anti-CND1), kindly provided by Drs. William Catterall and Ruth Westenbroek (University of Washington), were used at 1:250 dilution in blocking solution. Membranes were incubated in primary antibodies overnight at 4°C with constant agitation. The day after, membranes were washed 4× for 10 min in TBST and incubated with goat anti-rabbit HRP-conjugated secondary antibody (Bio-Rad, 1706515) at 1:10,000 in blocking solution for 1 h at RT with constant agitation. Membranes were washed in TBST, and bands were detected using Clarity Max Western ECL substrate (Bio-Rad, 1705062) and the iBright imaging system.

For NCX1 and caveolin-3 immunoblots, protein concentration was determined using a Pierce BCA Protein Assay Kit (Thermo, 23225) following the manufacturer's instructions. 25 µg of protein were denatured and resolved on Mini-Protean TGX Precast 4–15% Bio-Rad gels alongside Spectra Multicolor ladder (Thermo, 26634) using the same conditions as above. Proteins were transferred onto 0.2 µm PVDF membranes using a Trans-Blot Turbo Mini PVDF Transfer Pack (Bio-Rad, 1704156) on a Trans-Blot Turbo Transfer System (Bio-Rad) at 25 V and 1.3 A for 14 min. Membranes were stained for total protein with Ponceau S. For immunoblotting, membranes were blocked in 5% milk in TBST at RT for 1 h, then incubated overnight at 4°C with constant agitation in the appropriate primary antibody diluted in blocking solution: mouse monoclonal anti-NCX1 (GeneTex, GTX22869) at 1:200, or rabbit polyclonal anti-caveolin-3 (Abcam, ab2912) at 1:500. Membranes were washed 4× for 10 min in TBST and incubated for 1 h at RT with constant agitation in the appropriate fluorescent secondary antibody at 1:15,000 in blocking solution: IRDye 800CW goat anti-mouse (LI-COR, 926-32210) for anti-NCX1, or IRDye 680RD goat anti-rabbit (LI-COR, 926-68071) for anti-caveolin-3. After washing in TBST, membranes were imaged on a LI-COR Odyssey M imaging system.

Protein abundance was calculated by measuring the area under the curve of the band intensity profile using ImageJ (NIH). Each band was normalized to total protein and reported relative to the abundance in young pacemaker samples within the same experiment.

Electrophysiology

The composition of all the solutions used for electrophysiology can be found in the Solutions section. Recordings of single channels and whole-cell currents were performed at room temperature. Calcium currents were recorded using the whole-cell configuration of the patch-clamp technique in voltage-clamp mode. Isolated pacemaker cells were perfused with Tyrode’s III solution. Borosilicate patch pipettes with resistances of 3–6 MΩ were used. Once the gigaseal was formed, the Tyrode’s III bath solution was exchanged for the I_{Ca} external solution. Current–voltage relationships were obtained by applying a

series of 20-ms depolarizing pulses from a holding potential of -75 mV to test potentials ranging from -75 to +75 mV at a 5 mV interval. The voltage dependence of channel activation (G/Gmax) was obtained from the resultant currents by converting them to conductances using the equation, G = I_{Ca}/ (test pulse potential – reversal potential of I_{Ca}); normalized G/Gmax was plotted as a function of test potential. Currents were sampled at 10 kHz and low pass filtered at 2 kHz using an Axopatch 200B amplifier. To isolate the L-type and T-type components of the calcium current, after recording total I_{Ca}, the bath solution was exchanged for external solution containing 10 μM nifedipine (Millipore, 481981).

Recordings of action potentials were performed at 32-34˚C. Spontaneous pacemaker action potentials were recorded in current-clamp mode using the perforated-patch configuration. On the day of the experiment, freshly prepared β-Escin was added to the intracellular solution to reach a final concentration of 25 μM. Borosilicate patch pipettes with resistances of 3–6 MΩ were used. Spontaneous activity was recorded in the gap-free mode with no holding or transient current applied. Data was sampled at 10 kHz and low pass filtered at 2 kHz using an Axopatch 200B amplifier. Action potential parameters were analyzed manually using pCLAMP 11 (Molecular Devices). For Bay K experiments, the external solution was supplemented with 500 nM of Bay K8644.

Single-channel activity was recorded from cell-attached patches. Borosilicate patch pipettes with resistances of 5–7 MΩ were used. Once the gigaseal was obtained, the Tyrode's III bath solution was exchanged for the single-channel recording external solution. Cells were stimulated with 50 sweeps of a 200 ms square voltage pulse from a resting membrane potential of -80 mV to -30 mV. Current traces were sampled at 10 kHz and low pass filtered at 2 kHz using an Axopatch 200B amplifier. Currents were analyzed using pCLAMP 11 (Molecular Devices). The single-channel event detection algorithm of pCLAMP was used to measure single-channel opening amplitudes and times.

Calcium photometry and measurement of SR calcium load

A 100 µl drop of isolated pacemaker cells was placed on a coverslip and loaded with the fluorescent calcium indicator Cal-520 AM (1.5 µM) in KB solution for 10 minutes at room temperature, then constantly perfused with Tyrodes III solution and allowed 10 more minutes for de-esterification before imaging. Cells were then perfused with calcium-free Tyrode's solution to eliminate extracellular calcium influx. To induce rapid sarcoplasmic reticulum (SR) calcium release, a localized 20 mM caffeine puffs was applied using a glass micropipette positioned near the cell with a Picospritzer. Fluorescence signals were recorded using an IONOPTIX photometry system. Data was analyzed using IONOPTIX software and Igor Pro 9. For Bay K experiments, the calcium-free solution contained 500 nM of Bay K 8644.

Immunocytochemistry

For immunostaining $Ca_V1.2$ and $Ca_V1.3$ channels in pacemaker cells, cells were isolated and plated on PLL-coated coverslips for 2 hours to attach. Excess solution was removed carefully, and cells were fixed with 4% PFA in PBS for 15 min at RT. Unless stated otherwise, all the washing steps in this protocol consisted of 3 rinses with PBS followed by 3 x 5 min washes with PBS on a rocker at low speed. After washing with PFA with PBS, cells were incubated with 50 mM Glycine in PBS for 15 min at RT (aldehyde reduction) and then washed again with PBS. Cells were blocked with 3% w/v BSA - 0.25% v/v Triton X-100 in PBS (blocking solution) for 1h at RT. Primary antibodies were diluted in blocking solution to a concentration of 10 µg/mL (100 µl/coverslip) and incubated overnight at 4ºC under gentle orbital agitation. Cells were immunostained using the following antibodies: rabbit polyclonal anti-$Ca_V1.2$ (anti-CNC1), rabbit polyclonal $Ca_V1.3$ (anti-CND1), guinea pig anti-HCN4 (Alomone, APC-052-GP), mouse monoclonal anti-NCX1 (GeneTex, GTX22869), rabbit polyclonal anti-caveolin-3 (Abcam, ab2912), mouse caveolin-3 (BD Biosciences, 610421). Anti-CNC1 and anti-CND1 were kindly provided by Drs. William Catterall and Ruth Westenbroek (University of Washington). After primary antibody incubation, cells were washed with PBS and incubated for 1 h at RT in blocking solution with Alexa Fluor-conjugated secondary antibodies at 2 µg/ml. The specific secondary antibody was selected to match the host species of each primary and the

fluorophore required for the imaging modality. Secondary antibodies used included donkey anti-rabbit Alexa Fluor 647 (Invitrogen, A32795), donkey anti-mouse Alexa Fluor 647 (Invitrogen, A32787), donkey anti-rabbit Alexa Fluor 555 (Invitrogen, A32794), donkey anti-mouse Alexa Fluor 555 (Invitrogen, A32773), and goat anti-guinea pig Alexa Fluor 488 (Invitrogen, A11073). For super-resolution microscopy (STORM), Alexa Fluor 647-conjugated secondaries were used.

Primary antibody specificity was assessed through a combination of published validation, internal positive controls, and negative controls. The rabbit polyclonal anti-$Ca_V1.2$ (anti-CNC1) and anti-$Ca_V1.3$ (anti-CND1) antibodies, generated in the Catterall laboratory, recognize distinct intracellular epitopes of each isoform and have been previously validated using heterologous expression systems and staining of tissues with known expression profiles[37,66,68,79]. Commercial antibodies against HCN4 (Alomone APC-052-GP and Millipore AB5808), NCX1 (GeneTex GTX22869), and caveolin-3 (Abcam ab2912; BD Biosciences 610421) have been widely used in cardiac tissue and show characteristic single bands at the expected molecular weights on immunoblots. As a negative control for immunostaining, parallel samples were processed with secondary antibody alone (primary antibody omitted); these preparations showed no detectable signal above background. Co-labeling with HCN4, a cardiac pacemaker cell marker localized to the plasma membrane, served as an internal positive control to corroborate cell identity and to label the plasma membrane. Within individual experiments, all imaging was performed with identical acquisition settings across experimental and control conditions. Representative images shown in the figures were selected to reflect the mean of the quantified parameter; extreme examples were avoided.

Cell imaging

For Airyscan and TIRF microscopy, immunostained pacemaker cells were mounted in ProLong mounting medium on #1.5 glass coverslips and imaged. We used a Zeiss LSM 880 confocal microscope equipped with an Airyscan detector and a 63×/1.4 NA oil-immersion objective. For experiments focused on the footprint plane, pacemaker cells were identified and their plasma membrane labeled using HCN4 immunostaining.

Airyscan imaging was performed using definite focus control to maintain the focal plane across sequential channel acquisitions. For each cell, the equatorial plane was first identified as the mid-cell optical section where HCN4 outlined the entire cell as a ring-like structure. The footprint plane was defined as the basal optical section in which HCN4 formed a compact, homogeneous region corresponding to the cell–coverslip contact area. The HCN4 signal was used to select the footprint plane, and the fluorescent LTCC signal within the same plane was then acquired. All quantitative LTCC fluorescence measurements were performed exclusively on this footprint plane. For footprint plane validation, wheat germ agglutinin (WGA- fluorescein)-labeled cells were imaged using the same Airyscan configuration and, in parallel experiments, on a Leica DMi8 inverted microscope equipped for total internal reflection fluorescence (TIRF) microscopy with a 100× objective and a 110 nm penetration depth.

For super-resolution microscopy, coverslips were mounted on microscope slides with a round cavity (NeoLab Migge Laborbedarf-Vertriebs GmbH, Germany) using MEA-GLOX imaging buffer and sealed with Twinsil (Picodent, Germany). The imaging buffer contained 10 mM MEA, 0.56 mg/ml glucose oxidase, 34 µg/ml catalase, and 10% w/v glucose in TN buffer (50 mM Tris-HCl pH 8, 10 mM NaCl). A super-resolution ground-state depletion system (SR-GSD, Leica) based on stochastic single-molecule localization was used to generate super-resolution images of $Ca_V1.2$ and $Ca_V1.3$ channels in pacemaker cells. The Leica SR-GSD system was equipped with 642 nm high-power lasers (2.1 kW/cm2) and a 405 nm 30 mW laser. Images were obtained using a 160X HCX Plan-Apochromat (NA 1.43) oil-immersion lens and an EMCCD camera (iXon3 897; Andor Technology). For all experiments, the camera was running in frame-transfer mode at a frame rate of 100 Hz (10 ms exposure time). Fluorescence was detected through Leica high-power TIRF 642 HP-T filter cube with an emission band-pass filter of 660–760 nm. The resolution of the microscope is 20 nm lateral and 50 nm axial, ensuring the imaging of channels at the plasma membrane. Super-resolution localization images of $Ca_V1.2$ and $Ca_V1.3$ channel distribution were reconstructed using the coordinates of centroids obtained by fitting single-molecule fluorescence signals with a 2D Gaussian function using LASAF software (Leica). A total of 50,000 images were used to construct

the images. To disrupt caveolae, freshly isolated young pacemaker cells were incubated in Tyrode buffer containing 5 mM methyl-β-cyclodextrin (MβCD; Sigma-Aldrich, C4555) at room temperature for 30 minutes. Control cells from the same isolation were incubated in Tyrode buffer without MβCD for the same duration. After treatment, cells from both conditions were prepared for super-resolution imaging.

Study design and group sizes

Sample sizes were selected based on variance observed in prior experiments of a similar nature and are comparable to those used in published studies of sinoatrial node pacemaker cell function. No formal power calculation was performed. Animals were not randomized or blinded to group allocation. Blinding was not possible because young and old animals are visibly different. We acknowledge this as a potential source of bias. Exclusion criteria were defined a priori and included overt illness. All animals underwent necropsy after tissue collection to identify comorbidities that could confound interpretation, such as tumors. Datasets were evaluated for outliers using the ROUT method (Q = 1%) in GraphPad Prism 10.

Statistical Analysis

Data were analyzed in GraphPad Prism 10 unless otherwise noted. Normality of each dataset was assessed using the Shapiro-Wilk test. For analyses involving multiple cells from the same animal, we accounted for the hierarchical structure of the data by treating cells as nested within animals. We prioritized modeling this dependency over the assumption of normality because violating independence of clustered observations introduces substantial bias, whereas t-based tests are robust to moderate deviations from normality when statistical inference is made at the animal level. For nested comparisons between two groups, we therefore used nested t-tests. For Figure 7 panels B, F, G, and H, we used a linear mixed effects model fitted with the lme4 package in R (value ~ age × condition + (1|mouse/cell_id)), with age and treatment as fixed effects and cell nested within mouse as a random effect. This model accounted for both the paired repeated-measures design and the non-independence of cells from the same animal. Post-hoc comparisons used Sidak correction for 4 pre-specified contrasts. For Western blot data,

where each point represented a single animal and N was below 10 per group, two-tailed Mann-Whitney tests were used.

The specific statistical test used for each analysis is indicated in the corresponding figure legend. No experiment-wide multiple-testing correction was applied; p-values are reported without formal adjustment for multiple comparisons. A threshold of $p < 0.05$ was considered statistically significant. Results are reported as mean ± SEM unless otherwise noted, and the number of cells and animals for each analysis is reported in the figure legends.

SUPPLEMENTARY ONLINE FIGURES

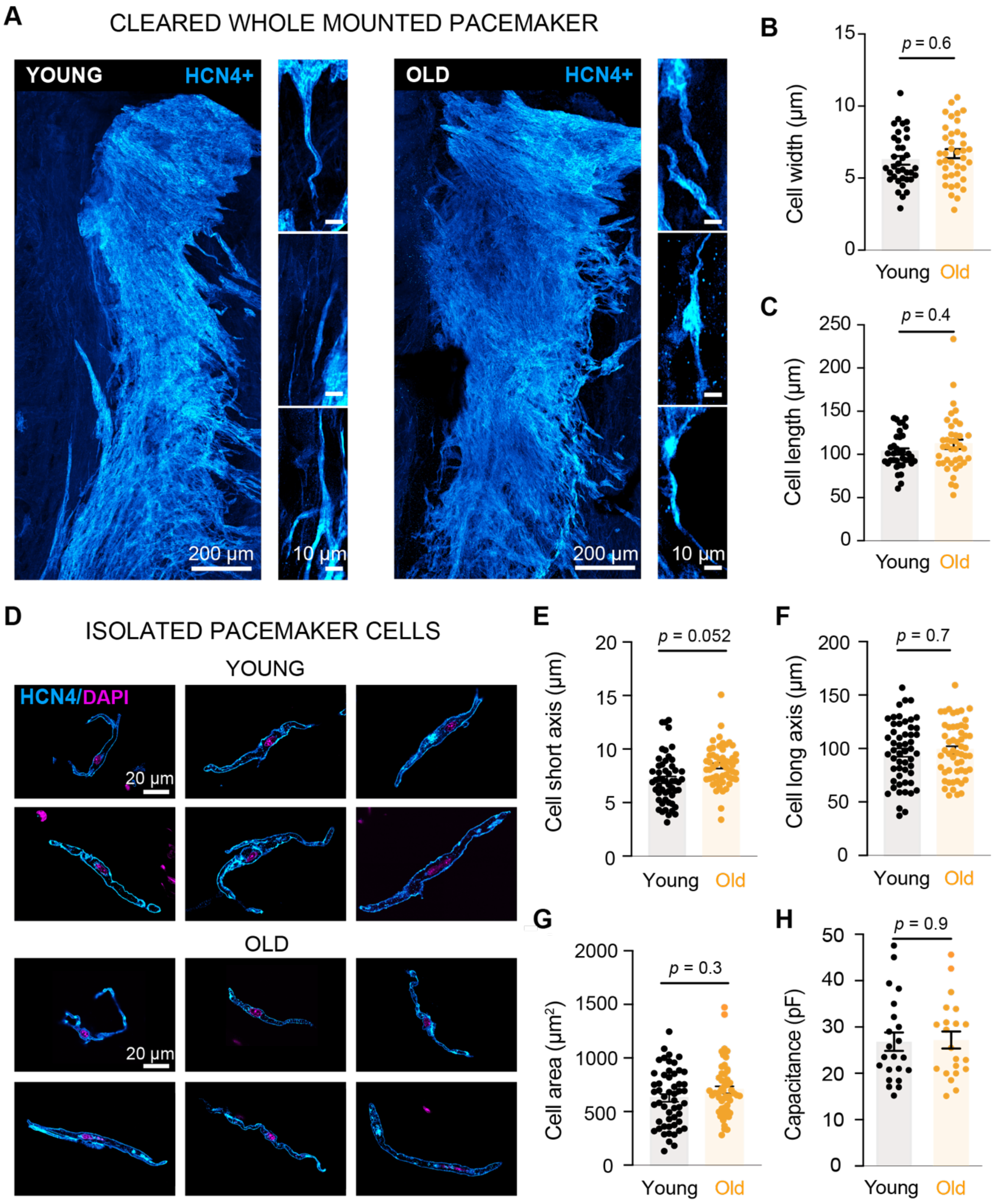

Online Figure S1. Aging is not associated with detectable changes in pacemaker cell size. A. Pictures of heart explants showing the pacemaker region stained against HCN4 in young (left) and old (right) mice. **B.** Comparison of cell width from HCN4-positive cells between young and old animals measured from tissue explants. **C.** Comparison of cell length from HCN4-positive cells between young and old animals measured from tissue explants. Sample numbers were n = 37 cells, N = 3 mice for the young group, and n = 39 cells,

N = 3 mice for the old. **D.** Representative images of isolated pacemaker cells from young (top) or old (bottom) mice stained against HCN4. Comparison of short axis (**E**), long axis (**F**), and cell area (**G**) from isolated HCN4-positive cells between young and old animals. Sample numbers were n = 53 cells, N = 5 mice for the young group, and n = 54 cells, N = 5 mice for the old. **H.** Comparison of the capacitance of young and old pacemaker cells. Sample numbers were n = 22 cells, N = 4 mice for the young group, and n = 21 cells, N = 4 mice for the old. Bars represent the mean and error bars the SEM. Statistical comparisons in panels B, C, and E-H used a nested t-test to account for cells nested within animals.

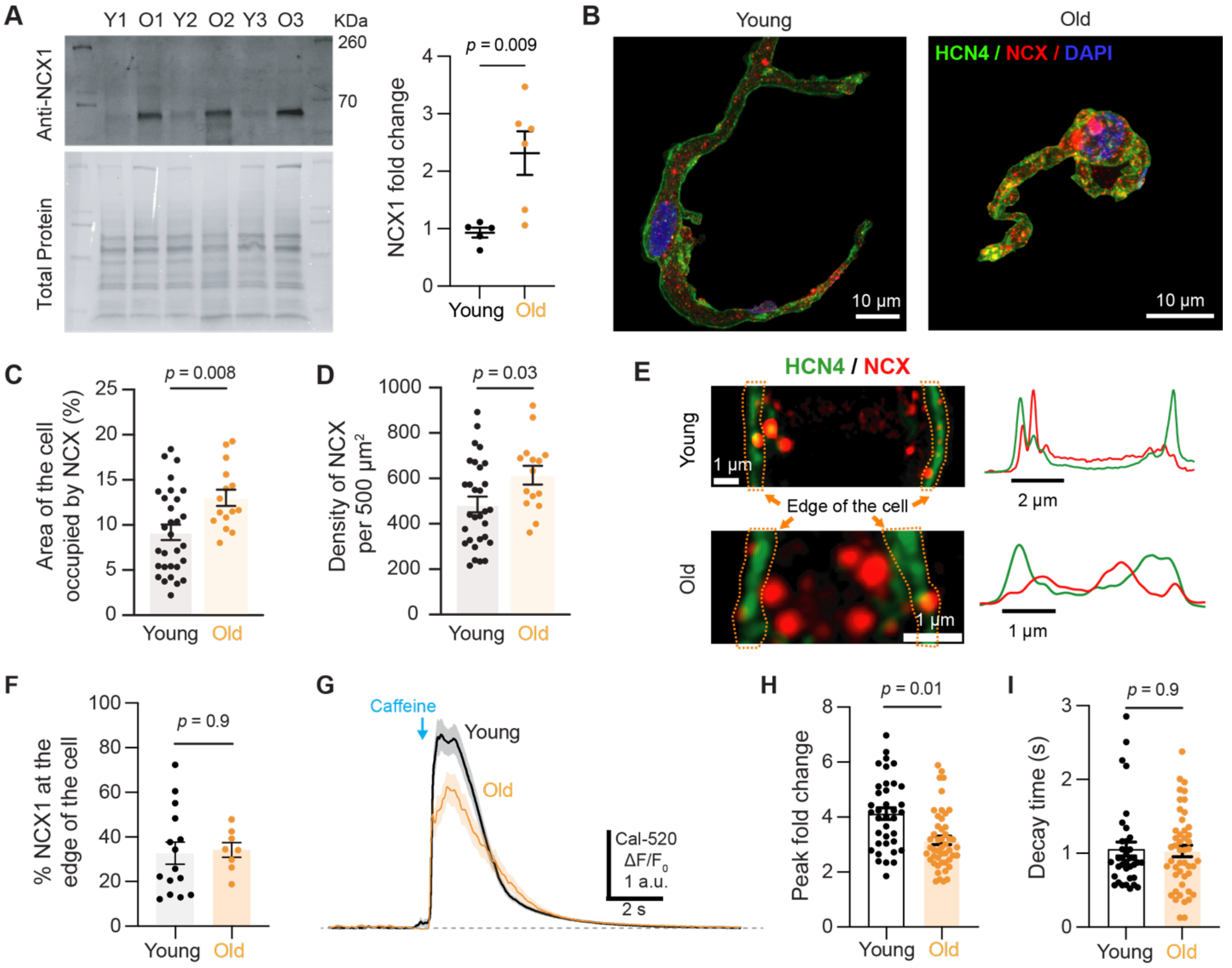

Online Figure S2. Reduction in L-type calcium current in old pacemaker cells is not associated with detectable changes in the calcium clearance mechanism. A. Western blot against NCX1 and total protein stain from pacemaker lysates from young and old mice (left). Fold change of NCX1 expression in old explants relative to young (right). Expression was normalized to total protein, and each data point represents the lysate from one animal (N = 5 young, N = 6 old). Statistical comparison used a non-parametric Mann-Whitney test comparing measurements from young and old samples. **B.** Representative AiryScan high-resolution images of young and old pacemaker cells labeled against HCN4 (green), NCX1 (red), and DAPI (blue). **C.** Comparison of percentage of the cell area occupied by NCX1 signal between young (n = 30 cells, N = 3 animals) and old (n = 15 cells, N = 3 animals). **D.** Comparison of NCX1 particle density found in young (n = 30 cells, N = 3 animals) and old (n = 15 cells, N = 3 animals). **E.** Representative AiryScan high-resolution images of cells labeled against HCN4 (green) and NCX1 (red) showing NCX1 signal at the plasma membrane (as indicated by the presence of HCN4) in young and old cells. (Right) Line scans showing the detection of NCX1 particles in the same region as the plasma membrane. **F.** Comparison of the percentage of area of the plasma membrane (PM) occupied by NCX1 in young (n = 15, N = 3) and old (n= 8, N = 3) cells. **G.** Average calcium transients induced by the fast application of 20 mM caffeine in young (black) and old (orange) cells. **H.** Comparison of the calcium transient amplitude between young (n = 37 cells, N = 3 animals) and old (n = 48 cells, N = 4 animals). Amplitude was measured as fold change of the peak relative to the basal level. **I.** Comparison of the decay time between young (n = 36 cells, N = 3 animals) and old (n = 47 cells, N = 4 animals). Decay time

was measured as the time constant (tau) of a single exponential function fitted to the phase of decay of the calcium transient. In all the scattered plots, bars represent the mean, and error bars the SEM. Statistical comparisons in panels C, D, F, H, and I used a nested t-test to account for cells nested within animals.

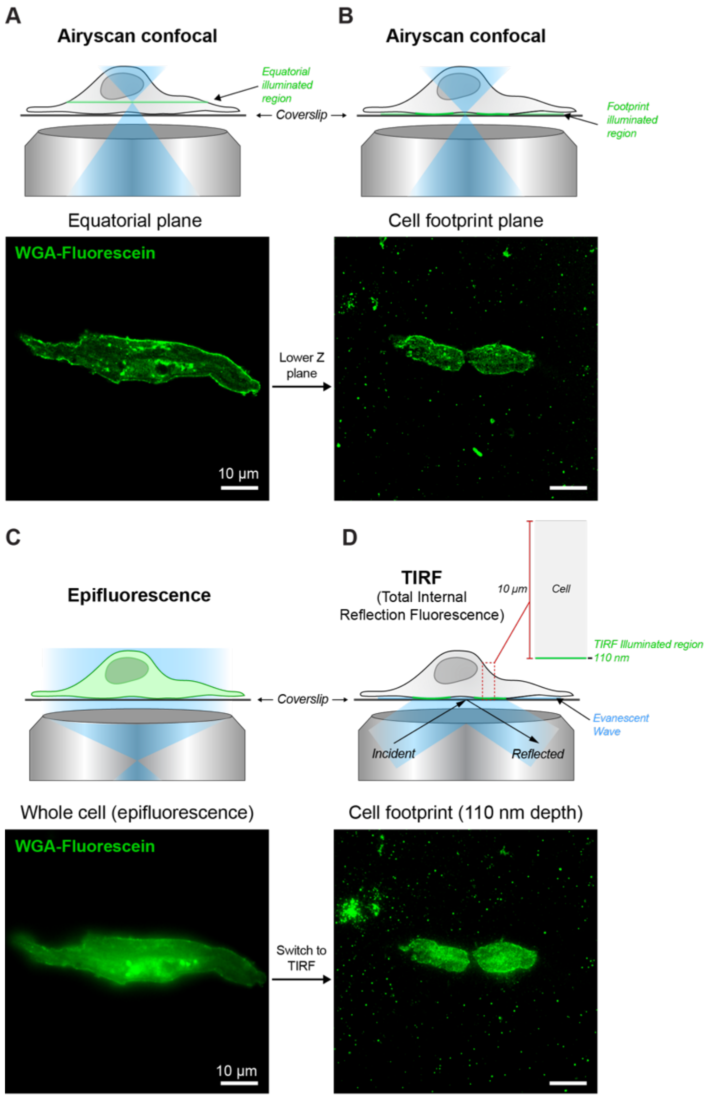

Online Figure S3. Airyscan footprint plane validation with WGA and TIRF microscopy. A. (Top) Schematic of Airyscan optical section at the equatorial plane of a pacemaker cell, only the middle plane of the cell is illuminated. (**Bottom**) Representative image of WGA-fluorescein staining at the equatorial plane showing the plasma membrane around cell perimeter. **B. (Top)** Schematic of Airyscan optical section at the footprint plane, only the footprint plane is illuminated. **(Bottom)** Representative image of the same WGA-labeled cell at the footprint showing compact WGA signal at adherent membrane region. **C. (Top)** Schematic of epifluorescence imaging where the whole cell is illuminated. **(Bottom)** Representative epifluorescence image of the same WGA-labeled cell showing whole-cell fluorescence distribution. **D. (Top)** Schematic of the TIRF principle, sample is illuminated at a critical angle to reflect the light, creating an evanescent field that penetrates ~110 nm from the coverslip. TIRF selectively excites the basal region of the cell that is in contact with the coverslip. **(Bottom)** Representative image of the same WGA-labeled cell in TIRF. Note that the WGA footprint morphology precisely matches the Airyscan footprint plane.

UNCROPPED WESTERN BLOTS

Unedited immunoblots against Ca_V1.2 and Ca_V1.3 channels used for the analysis in Figure 4. Y (young), O (old), numbers represent animal replicates. Dotted lines represent where membranes were cut.

Experiment 1

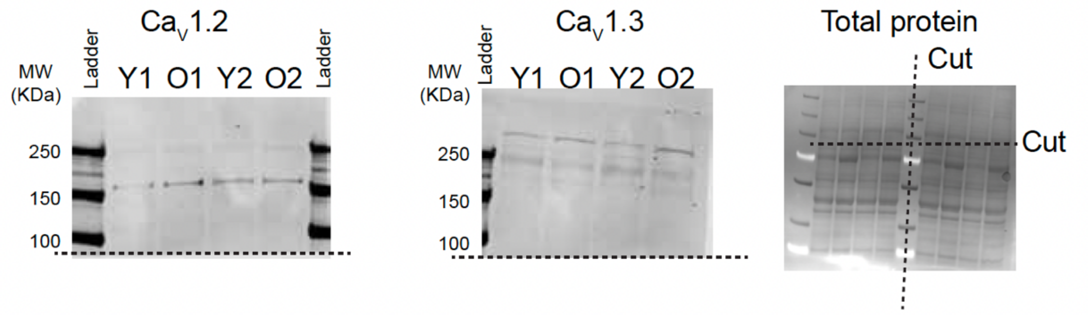

Experiment 2

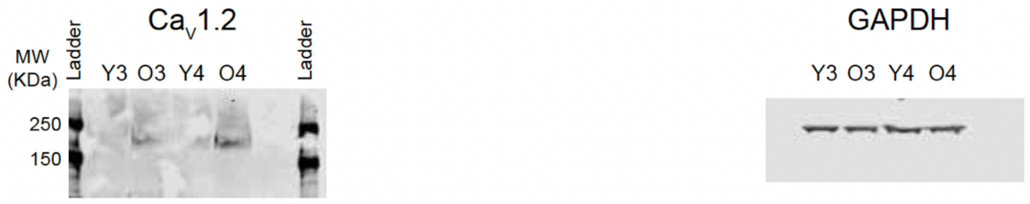

Experiment 3

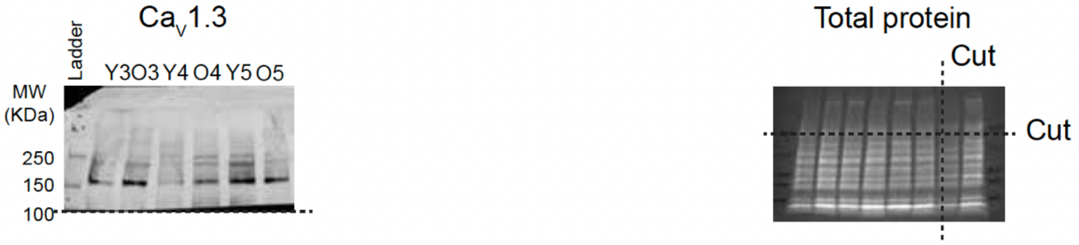

Unedited immunoblots against NCX1 used for the analysis in Online Figure S2. Y (young), O (old), numbers represent animal replicates. Dotted lines represent where membranes were cut.

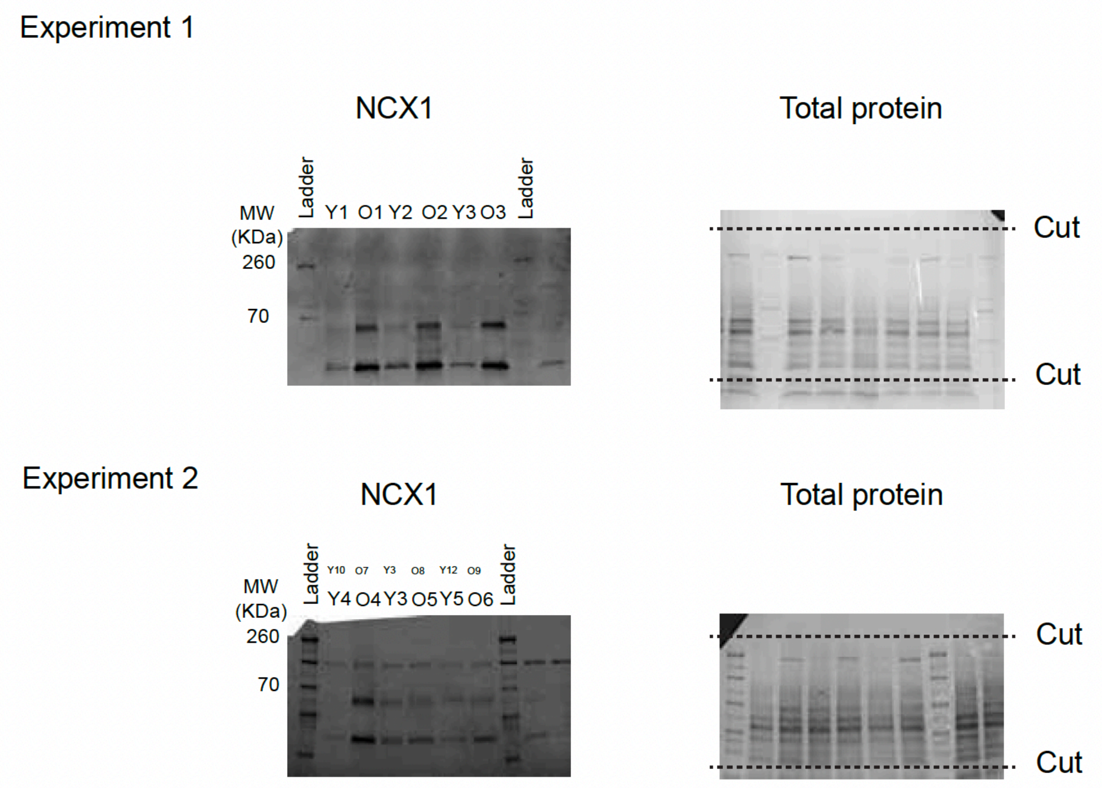

Unedited immunoblots against caveolin-3 used for the analysis in Figure 8. Y (young), O (old), numbers represent animal replicates. Dotted lines represent where membranes were cut.

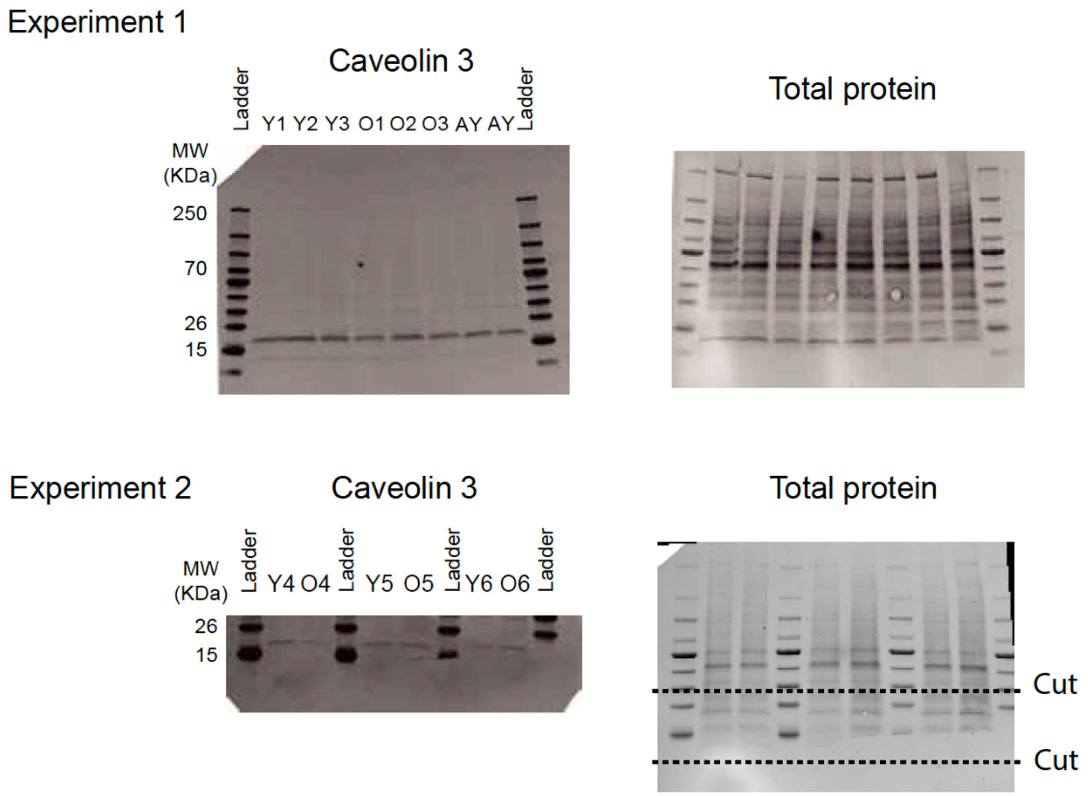

Major Resources Table

In order to allow validation and replication of experiments, all essential research materials listed in the Methods should be included in the Major Resources Table below. Authors are encouraged to use public repositories for protocols, data, code, and other materials and provide persistent identifiers and/or links to repositories when available. Authors may add or delete rows as needed.

Mouse Models Used in This Study

Strain	Vendor or Source	Background Strain	Sex	Other Information (e.g. breeding scheme for F1, F2…if applicable)	Persistent ID / URL
C57BL/6J	JAX (Strain #000664)	C57BL/6J	M	Ages 4-6 months	https://www.jax.org/strain/000664
C57BL/6JN	NIA/Charles River Lab	C57BL/6JN	M	Ages 24-30 months	https://www.nia.nih.gov/research/dab/aged-rodent-colonies

Additional Species Used in This Study (if applicable)

Strain	Vendor or Source	Background Strain	Sex	Other Information (e.g. breeding scheme for F1, F2…if applicable)	Persistent ID / URL
N/A					

Antibodies

Target antigen	Vendor or Source	Catalog #	Working concentration	Lot # (preferred but not required)	Persistent ID / URL
Ca_V1.2-CNC1	Drs. William Catterall and Ruth Westenbroek	N/A	10 µg/mL	N/A	N/A
Ca_V1.3-CND1	Drs. William Catterall and Ruth Westenbroek	N/A	10 µg/mL	N/A	N/A
HCN4	Alomone	APC-052-GP	10 µg/mL	APC052GP AN0402	https://www.alomone.com/p/anti-hcn4/APC-052-GP
HCN4	Millipore	AB5808	10 µg/mL		https://www.sigmaaldrich.com/US/en/product/mm/ab5808
NCX1	GeneTex	GTX22869	10 µg/mL	822502581	www.genetex.com/Product/Detail/NCX1-antibody-C2C12/GTX22869
Caveolin-3	Abcam	ab2912	10 µg/mL		https://www.abcam.com/en-us/products/ab2912
Caveolin-3	BD Biosciences	610421	10 µg/mL	1060059	https://www.bdbiosciences.com/en-us/products/reagents/western-blotting-and-molecular-reagents/western-blot-reagents/purified-mouse-anti-caveolin-3.610421?tab=product_details
Donkey anti-rabbit Alexa-647	Invitrogen	A32795	2 µg/ml	3234330	https://www.thermofisher.com/antibody/product/A-31573
Donkey anti-mouse Alexa-647	Invitrogen	A32787	2 µg/ml	ZI397339	https://www.thermofisher.com/antibody/product/A-31571

Donkey anti-Rabbit Alexa-555	Invitrogen	A32794	2 µg/ml	TH271030	https://www.thermofisher.com/antibody/product/A-31572
Donkey anti-mouse Alexa-555	Invitrogen	A32773	2 µg/ml	AC410633	https://www.thermofisher.com/antibody/product/A-31570
Goat anti-guinea pig Alexa-488	Invitrogen	A11073	2 µg/ml	2674373	https://www.thermofisher.com/antibody/product/A-11073
Goat anti-rabbit HRP	Bio-Rad	1706515	1:10,000	N/A	https://www.bio-rad.com/en-us/sku/1706515-goat-anti-rabbit-igg-h-l-hrp-conjugate
Goat anti-mouse IRDye 800CW	LI-COR Biosciences	926-32210	1:15,000	N/A	https://www.licor.com/bio/reagents/irdye-800cw-goat-anti-mouse-igg-secondary-antibody
Goat anti-rabbit IRDye 680RD	LI-COR Biosciences	926-68071	1:15,000	N/A	https://www.licor.com/bio/reagents/irdye-680rd-goat-anti-rabbit-igg-secondary-antibody

Drugs/Chemicals

Resource	Source	Cat #	Concentration
Methyl-β-cyclodextrin (MβCD)	Sigma-Aldrich	C4555	5 mM
Nifedipine	Millipore	481981	10 µM

DNA/cDNA Clones

Clone Name	Sequence	Source / Repository	Persistent ID / URL
N/A			

Cultured Cells

Name	Vendor or Source	Sex (F, M, or unknown)	Persistent ID / URL
N/A			

Data & Code Availability

Description	Source / Repository	Persistent ID / URL
N/A		

ARRIVE GUIDELINES

The ARRIVE guidelines (https://arriveguidelines.org/) are a checklist of recommendations to improve the reporting of research involving animals. Key elements of the study design should be included below to better enable readers to scrutinize the research adequately, evaluate its methodological rigor, and reproduce the methods or findings.

Study Design

**Groups**	**Sex**	**Age**	**Number (prior to experiment)**	**Number (after termination)**	**Littermates (Yes/No)**	**Other description**
Group 1 (Control-Young)	M	4-6 months	3	3-4	No	
Group 2 (Experimental-Old)	M	24-30 months	3	3-4	No	
Add more if needed						

Sample Size: Please explain how the sample size was decided. Please provide details of any a *prior* sample size calculation, if done.

Sample sizes were selected based on variance observed in prior experiments of a similar nature and are comparable to those used in published studies of sinoatrial node pacemaker cell function. No formal power calculation was performed

Inclusion Criteria

No formal inclusion criteria were specified *a priori*. All animals of the appropriate genotype, age, and sex that met general health standards were eligible for inclusion.

Exclusion Criteria

Exclusion criteria were defined a priori and included overt illness. All animals underwent necropsy after tissue collection to identify comorbidities that could confound interpretation, such as tumors. Datasets were evaluated for outliers using the ROUT method (Q = 1%) in GraphPad Prism 10.

Randomization and blinding:

Animals were not randomized or blinded to group allocation. Blinding was not possible because young and old animals are visibly different. We acknowledge this as a potential source of bias
